# Chaperone-Mediated Regulation of Tau Phase Separation, Fibrillation, and Toxicity

**DOI:** 10.1021/jacs.5c01369

**Published:** 2025-06-30

**Authors:** Cecilia Mörman, Axel Leppert, Giusy Pizzirusso, Zihan Zheng, Xun Sun, Rakesh Kumar, Henrik Biverstål, Michael Landreh, Jan Johansson, Luis Enrique Arroyo-Garcia, Jinghui Luo, Gefei Chen, Axel Abelein

**Affiliations:** † Department of Medicine Huddinge, 27106 Karolinska Institutet, Huddinge, 141 52 Huddinge, Sweden; ‡ Center for Life Sciences, 28498 Paul Scherrer Institute, Villigen, 5232 Villigen, Switzerland; § Department of Cell and Molecular Biology, Uppsala University, 75124 Uppsala, Sweden; ∥ Department of Microbiology, Tumor and Cell Biology, Karolinska Institutet, Solna, 171 65 Stockholm, Sweden; ⊥ Department of Neurobiology, Care Sciences and Society, Division of Neurogeriatrics, Center for Alzheimer Research, Karolinska Institutet, Solna, 171 77 Stockholm, Sweden; # Department of Women’s and Children’s Health, Karolinska Institutet, Solna, 171 77 Stockholm, Sweden; ¶ Department of Pharmacology, Xi’an Jiaotong University, 710061 Xi’an, Shaanxi, China; ∇ Department of Neurobiology, Care Sciences and Society, Division of Clinical Geriatrics, Center for Alzheimer Research, Karolinska Institutet, Huddinge, 141 83 Stockholm, Sweden

## Abstract

Biological condensates are involved in several essential processes but may also be tangled into disease progression in protein misfolding diseases such as Alzheimer’s disease and tauopathies. One hallmark of these disorders is the appearance of fibrillar aggregates formed by microtubule-stabilizing Tau protein. Notably, Tau can also assemble into biological condensates and droplets via liquid–liquid phase separation (LLPS). The molecular mechanisms of the conversion of functional Tau toward insoluble fibrils, potentially via LLPS processes, remain largely unknown, and efficient treatment approaches to target toxic pathways and species are still missing. Here, we show that the molecular chaperone-like Bri2 BRICHOS domain efficiently inhibits full-length Tau fibril formation and subsequent neurotoxicity by specifically suppressing secondary nucleation processes. Further, at substoichiometric ratios, Bri2 BRICHOS modulates the potency of Tau to form droplets, incorporates into Tau droplets, and alters the dynamic behavior of Tau. In contrast, at superstoichiometric Bri2 BRICHOS ratios, Tau droplet formation is abolished. Finally, Bri2 BRICHOS reduces Tau fibril toxicity in electrophysiological experiments on hippocampal slice preparations. Taken together, Bri2 BRICHOS targets molecular processes related to protein misfolding, where our study provides molecular insights into how the inhibition of secondary nucleation pathways and modulated droplet formation are eventually linked to attenuated neurotoxicity.

## Graphical abstract

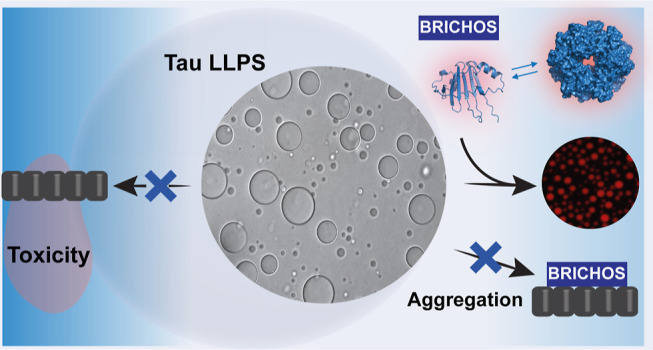

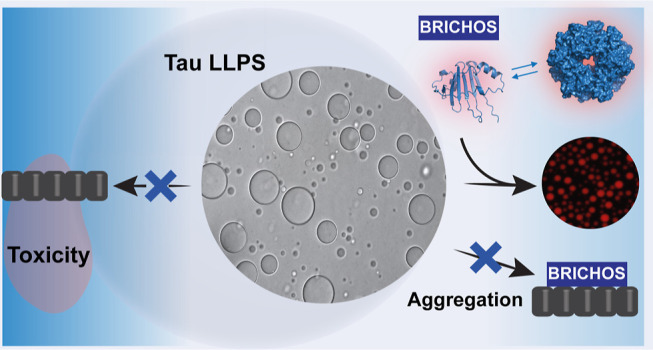

## Introduction

Self-assembly of proteins into ordered structures is highly relevant for biological function but is also associated with a variety of diseases. These diseases, commonly referred to as protein misfolding diseases, include prominent examples of devastating and irreversible disorders, such as Alzheimer’s disease (AD), which are growing challenges as the global population ages.^1^ In the brains of AD patients, pathological proteinaceous deposits are found, in particular aggregated amyloid-β (Aβ) peptides in senile plaques and hyperphosphorylated and aggregated Tau protein in neurofibrillary tangles (NFTs).^2^ Tau is mainly expressed in neurons and exhibits one of its main functions as a microtubule-stabilizing protein.^3,4^ The primary structure consists of two acidic regions in the N-terminal part (N1 and N2), proline-rich domains (P1 and P2), a microtubule-binding domain (MTB) of four pseudorepeats (R1, R2, R3 and R4), and a C-terminal part. Tau is a monomeric and intrinsically disordered protein and may aggregate via nucleation processes, forming larger assemblies including oligomeric species and eventually insoluble fibrils as part of the pathogenesis.^5,6^ The Tau fibril structures comprise a core with a cross-β structure and an unstructured fuzzy coat.^7^ Tau protein also misfolds and aggregates in several other diseases besides AD, generally termed tauopathies,^8^ outlining Tau aggregation, in particular toxic nucleation pathways and/or species, as a potential common target for therapeutic agents for this class of diseases.^2^

During the last years, the importance of liquid–liquid phase separation (LLPS) processes and formation of biomolecular condensates via weak and multivalent interactions during the early stages of protein aggregation has been highlighted.^9–12^ Biomolecular condensates, or droplets, are collective names for membrane-less assemblies of biomolecules such as proteins and nucleic acids on the mesoscopic level.^9^ Tau protein readily forms dynamic liquid droplets in vitro and in cells.^10,13,14^ Full-length Tau, here referred to as Tau_441_, has higher LLPS propensity compared to other isoforms.^3^ At physiological pH, the net charge is around +3.1, with a pronounced negatively charged N-terminus and, overall, positively charged pseudorepeats, where the charge distribution is important for the occurrence of Tau condensation.^3^ Further, the disease-related mutations P301L and P301S display a similar or even higher tendency of LLPS as the wild-type Tau.^10,15^

The human proteostasis network is regulated and synchronized by ∼300 molecular chaperones present in all cell types.^16,17^ A gradual decline of proteostasis and impaired protein quality control are evident during aging.^17^ In the past, high-molecular-weight molecular chaperones like Hsp90, S100B, Hsp22, and peptidyl prolyl isomerase A (PPIA) have been studied as inhibitors of Tau protein aggregation.^7,18–24^ Another class of molecular chaperone-like proteins is endogenous protein domains evolved to protect aggregation-prone regions in the respective pro-proteins. One prominent example is the BRICHOS domain, which has been identified as part of a pro-protein in at least 13 protein families with overall low sequence identities but that share a similar fold and three conserved residues.^25,26^ In the context of neurodegenerative diseases, the BRICHOS domain from the Bri2 protein (encoded by the *ITM2B* gene) is highly relevant since it is expressed, among other organs, in the brain,^27^ and together with proSP-C BRICHOS, it has previously been found to inhibit amyloid formation by suppression of secondary nucleation processes of Aβ, IAPP, and α-synuclein by us and others.^28–33^ In addition, elevated levels and impaired activity of Bri2 in the hippocampus of AD patients have been reported.^34^ The assembly state of Bri2 BRICHOS (monomer, dimer, or oligomer) was identified as a determinant for targeting different misfolded protein species.^28^ Monomeric Bri2 BRICHOS, which can be stabilized by the R221E mutation,^35^ shows a preferential inhibitory effect on the formation of Aβ species related to neuronal toxicity, whereas oligomeric Bri2 BRICHOS also targets nonfibrillar protein aggregation.^28,36^ Recent treatment studies of AD mouse models by intravenous injections of recombinant Bri2 BRICHOS showed reduced plaque load and neuroinflammation with improved cognitive behavior, which can be related to the in vitro inhibitory effect and normalization of expression of plaque-induced genes.^37–40^ Based on the advances of these studies, in this project, the recombinant human, 121-residue-long, Bri2 BRICHOS domain including the mutation R221E was used.

To prevent Tau-associated neurodegenerative diseases, molecular insights into Tau dysfunction, potentially in combination with new therapeutic approaches, are highly demanded. Experimental in vitro and in vivo evidence suggests that aberrant Tau LLPS may be a precursor for aggregation,^14,41^ as droplets from several other proteins than Tau have been reported to mature into an insoluble, fibrillar state.^42–46^ However, clear causal links between protein LLPS and amyloid formation are still missing, as well as strategies for how to regulate these processes.^47^ Here, we studied the effect of Bri2 BRICHOS (Figure 1A) on Tau_441_ aggregation in relation to Tau_441_ LLPS processes and the associated Tau-induced toxicity. We found that the molecular chaperone Bri2 BRICHOS (1) modulates Tau protein phase separation and inhibits maturation of liquid-to-solid transitions, (2) inhibits the fibril generation by preventing secondary nucleation processes, and (3) suppresses Tau-induced toxicity measured in electrophysiological measurements. Taken together, the current study shows ways how a molecular chaperone could be utilized to safeguard healthy Tau protein conformations, which could be developed into future protein-based therapeutic strategies to combat protein misfolding diseases.

**Figure 1:**
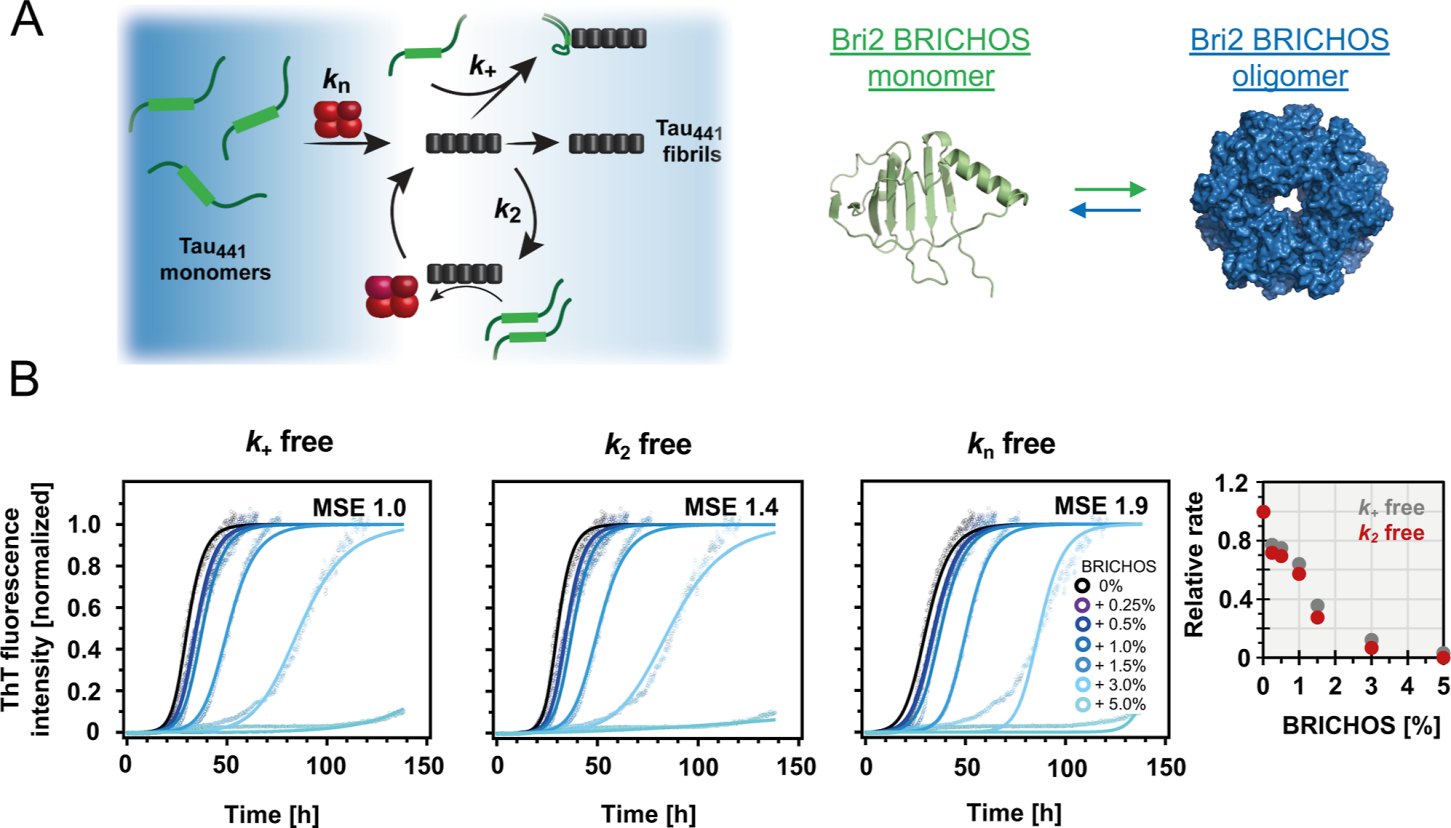
Bri2 BRICHOS inhibits cofactor-free Tau_441_ aggregation by inhibiting Tau_441_ secondary nucleation processes. (A) Schematic representations of Tau_441_ aggregation involving microscopic nucleation processes and modeled structures of Bri2 BRICHOS by AlphaFold2 ^58^ and cryo-EM data for the Bri2 BRICHOS oligomer, PDB ID 8RNU,^59^ visualized with PyMOL. (B) Global fit analysis of 20 μM Tau_441_ fibrillation in 20 mM NaP buffer, pH 7.4, with agitation at 37 °C, in the presence of oligomeric Bri2 BRICHOS using a secondary nucleation-dominated model,^51^ giving the best fits with *k*_+_ or *k*_2_ as the individual free fitting parameter. MSE refers to the relative mean square error from the fits.

## Experimental Section

### Expression and Purification of Recombinant Proteins

Expression and purification of recombinant full-length Tau_441_ protein and Bri2 BRICHOS were performed following previously described protocols.^31,35,48^ In brief, Tau_441_ was expressed and induced during 2 h with 0.4 mM IPTG in a BL21­(DE3) (*E.coli*) cell line. The Tau_441_ protein was purified by sonication of the resuspended cell pellet in 50 mM NaP buffer (pH 6.5), 1 mM EDTA, and one protease inhibitor cocktail tablet 1× (cOmplete, EDTA-free, Roche), followed by centrifugation to remove larger cell constituents from the proteins. The supernatant was heat-treated, followed by centrifugation to remove insoluble remains. The supernatant with the remaining soluble proteins was kept at −20 °C, thawed for further purification, and applied to cation-exchange chromatography (HiTrap SP HP, Cytiva), eluted by an increasing NaCl concentration gradient. The fractions were analyzed on SDS–PAGE, and the fractions containing pure Tau_441_ were pooled together. The NaCl was removed using a HiPrep 26/10 Desalting column (Cytiva) in 20 mM NaP, pH 7.4, with 0.2 mM EDTA, and Tau_441_ was aliquoted and stored at −20 °C. An extinction coefficient of 7550 M^–1^ cm^–1^ was used to determine the Tau_441_ protein concentration, and the purity and quality of Tau_441_ were controlled with mass spectrometry (Figure S1). ^15^N-labeled Tau_441_ protein was expressed similarly, but with the usage of minimal M9 media instead of LB media, and ^15^NH_4_Cl for the expression was added. The Tau P301L mutant was expressed as described previously.^49^

The BRICHOS domain in monomeric and oligomeric states from the Bri2 protein, constituting residues 113–231 of the Bri2 protein with two additional N-terminal residues (sequence of GSQ­TI_5_ EEN­IK_10_ IFE­EE_15_ EV­EFI_20_ SV­PVP_25_ EF­ADS_30_ DP­ANI_35_ VH­DFN_40_ KK­LTA_45_ YL­DLN_50_ LD­KCY_55_ VI­PLN_60_ TSI­VM_65_ PP­RNL_70_LE­LLI_75_ NI­KAG_80_ TY­LPQ_85_ SY­LIH_90_ EH­MVI_95_ TD­RIE_100_ NI­DHL_105_ GF­FIY_110_ EL­CHD_115_ KE­TYK_120_L), was expressed within the plasmid pT7NT*-Bri2 113-231 R221E (Addgene plasmid # 138134) in SHuffle T7 (K12) cells.^31^ In short, 0.5 mM IPTG was used for induction overnight at 20 °C, and the cells were harvested by centrifugation in 20 mM Tris–HCl (pH 8). The protein was purified by the following steps: sonication of the cells, centrifugation of the lysate, and supernatant loading on a Ni-NTA column (GE Healthcare Bio-Science), followed by a wash and elution with imidazole. The imidazole was removed by dialysis overnight in 20 mM Tris (pH 8.0) together with thrombin to remove the N-terminal tag (His6-NT*). The N-terminal tag was separated from the Bri2 BRICHOS by a Ni-NTA gravity column. Monomeric and oligomeric Bri2 BRICHOS were collected through size exclusion chromatography (SEC), and the concentrations were determined with an extinction coefficient of 9065 M^–1^ cm^–1^. The purity of the BRICHOS monomer and multimer samples was validated by SDS–PAGE. ^15^N-labeled Bri2 BRICHOS protein was expressed similarly, but with the usage of minimal M9 media instead of LB media, and ^15^NH_4_Cl for the expression was added.

Fluorescent labeling of Tau and Bri2 BRICHOS was performed with an amine-reactive Atto655 NHS-ester dye (Sigma-Aldrich, 76245) previously described in detail.^50^ All buffers and solvents used for the experiments were filtered and degassed before usage.

### Thioflavin T (ThT) Fluorescence Kinetics

To follow the fibrillation of Tau_441_ in the absence and presence of Bri2 BRICHOS, a Thioflavin T (ThT) assay was used. 15–20 μM Tau_441_ in 20 mM NaP (pH 7.4), and 0.2 mM EDTA supplemented with 50 μM ThT (λ_ex_: 440 nm, λ_em_: 480 nm) was used in 384-well plates (Corning), 20 μL per well, and fluorescence measured every 10 min in a FLUOStar Omega platereader (BMG Labtech) at 37 °C. To induce fibrillation without cofactors, extensive orbital shaking at 300 rpm was used with two glass beads with a diameter of 1 mm in each well for 9 min between the fluorescence readings. All conditions were repeated several times and measured with 6 replicates, giving a median value of six replicates and standard deviations. The ThT kinetic curves were analyzed with sigmoidal curve fitting, as well as with a global fit analysis applying a rate law with a secondary nucleation dominated model in the AmyloFit interface.^51^ Two of the three rate constants *k*_2_, *k*_*+*_, and *k*_*n*_ in the model were held constant, whereas the third was allowed to vary.

Seeded aggregation kinetics were performed with sonicated preformed seeds. Preformed seeds were prepared from sonication of fibrils from previous Tau_441_ fibrillation experiments; the sonication was executed for 3 min with 2 s on and 2 s off with 20% power amplitude with a probe sonicator (Vibra-Cell Sonics). The seed concentration was based on the initial monomeric protein concentration.

### Nuclear Magnetic Resonance (NMR)

The nuclear magnetic resonance (NMR) experiments were performed on a 700 MHz Bruker Avance spectrometer equipped with a cryogenic probe. Unlabeled monomeric/oligomeric Bri2 BRICHOS or Tau_441_ were titrated upon 100 μM monomeric ^15^N-Tau_441_ or 68 μM monomeric ^15^N-Bri2 BRICHOS in 20 mM NaP and 0.2 mM EDTA (pH 7.4), respectively, and used for 2D ^1^H–^15^N-HSQC titration experiments. Additional experiments were performed with 6% (w/vol) PEG8000 for LLPS conditions using 62 μM monomeric ^15^N-Tau_441_. The experiments were performed at 298 K with 90/10 H_2_O/D_2_O, and the experimental temperature was calibrated with an external thermometer. The spectra with and without BRICHOS, both in aqueous solution and during LLPS conditions, were compared by (1) calculating the relative intensities based on the amplitude intensities and (2) determining the chemical shift changes (CSC). The CSC values were calculated from eq 1:

1 $$\Delta \delta ={\left(\left({\left(\frac{\Delta \delta_{N}}{5}\right)}^{2}+{\left(\Delta \delta_{H}\right)}^{2}\right)/2\right)}^{1/2}$$

The cross-peak assignment of Tau_441_ (BMRB ID 50701) was achieved by a comparison to published work,^52^ and the monomeric Bri2 BRICHOS assignment has been performed in-house previously.^31^ Data were processed and analyzed with software’s Topspin v.4.2.0 (Bruker) and Poky^53^ (University of Colorado Denver).

### Circular Dichroism (CD)

Circular dichroism (CD) measurements were conducted on a J-1500 circular dichroism spectrophotometer (JASCO) by recording spectra in the spectral range of 190–260 nm in a 1 mm quartz cuvette at room temperature with a bandwidth of 0.5 nm, resolution/step size of 0.5 nm, and a scanning speed of 20 nm/min. Tau_441_ (5 μM) with and without 10 μM monomeric or oligomeric Bri2 BRICHOS in 20 mM NaP (pH 7.4), 0.2 mM EDTA, in the absence and presence of 5% (w/vol) PEG8000 were measured. The spectra displayed are the average of three consecutive scans with background subtraction.

### Dynamic Light Scattering (DLS)

Dynamic light scattering (DLS) measurements and data acquisition including analysis were performed on a Prometheus Panta instrument (Nanotemper) with a 660 nm laser at a 90*°* scattering angle, using sample capillaries with 20 μL of 25 μM Tau_441_ with and without equimolar concentration of monomeric or oligomeric Bri2 BRICHOS. NaP (20 mM) and 0.2 mM EDTA, pH 7.4, were used as a buffer for all samples. The samples were prepared fresh and measured directly. All stock solutions were centrifuged to remove any large contaminants and bubbles. The hydrodynamic radius of the samples was calculated from the autocorrelation functions from 10 runs of each sample condition. The measurements were repeated at least twice.

### Turbidity Measurements

The turbidity was measured in a POLARStar Omega platereader (BMG Labtech) in 96- or 384-well plates (Corning) of both fresh samples and over time at room temperature. The background absorbance was subtracted from the sample measurements, measured with 3–6 replicates, and presented as an average. The phase diagrams were obtained from turbidity measurements by varying the PEG8000 concentrations (0, 2, 4, 6, 8, 10, and 12% (w/vol)) and Tau_441_ protein concentrations (0, 1, 2, 3, 5, 10, 15, and 20 μM).

### Gel Electrophoresis

Native PAGE was performed on freshly prepared 25 μM Tau_441_ mixed with and without 12.5 and 25 μM monomeric or oligomeric Bri2 BRICHOS and added to a gel under nondenaturing conditions.

### Bright-Field Microscopy

Images on an EVOS FLoid Auto 2 (Invitrogen ThermoFisher Scientific) or an inverted Axio Observer microscope (Zeiss) were taken for freshly prepared samples and over time of 10–20 μM Tau_441_ mixed with several concentrations of monomeric or oligomeric Bri2 BRICHOS and 8% (w/vol) PEG8000 as an inducer of LLPS and performed in duplicates at room temperature. Tau_P301L_ (10 μM) was mixed with several concentrations of oligomeric Bri2 BRICHOS and 8% (w/vol) PEG8000. 96-well half-area low-binding plates (Corning) and a 20× water objective were used. Maturation of droplets was followed in the presence of 8% (w/vol) PEG8000 after incubation of 100 h. The images were analyzed by Fiji.

### Fluorescence Microscopy

Fluorescence microscopy imaging was performed using a Nikon Eclipse Ti series inverted microscope (Nikon) equipped with a Crest X-light V2 series confocal unit (Nikon), a Full Multiband Quad filter (FF01-440/5221/607/700, Em), and a Zyla sCMOS camera (Andor). Samples of 20 μL were imaged in 96-well half-area low-binding plates (Corning) after 45 min equilibration at room temperature in 20 mM NaP (pH 7.4), 0.2 mM EDTA, using a Plan Apo 40× objective (Nikon). Visualization of Bri2 BRICHOS incorporation into Tau_441_ droplets was performed with 20 μM Tau_441_ and 8% (w/vol) PEG8000 and 0.2 μM monomeric Bri2 BRICHOS-Atto655 or 0.2 μM NT*-Atto655 as a control (laser λ_ex_ 640 nm). NT* was expressed and purified as previously described.^54^

### Fluorescence Recovery after Photobleaching (FRAP)

To elucidate the fluidity of Tau_441_ droplets in the presence of Bri2 BRICHOS, fluorescence recovery after photobleaching (FRAP) experiments were performed using an LSM980-Airy microscope (Zeiss) equipped with an Airy detector 2 using a C-Apochromat 40×/1.2 water objective on samples of 10 μM Tau_441_ supplemented with 1% fluorescently labeled Tau441-atto655 with 8% (w/vol) PEG8000 in 20 mM sodium phosphate, 0.2 mM EDTA, pH 7.4. Oligomeric Bri2 BRICHOS (0, 5, or 10 μM) was added. A circular region of 1 μm diameter was used for photobleaching during 1 ms spot bleach with an 80% intensity 639 nm laser. The pinhole diameter was set to 47 μm. Seven different droplets were bleached for each condition, and data are represented as average and standard deviation.

### Toxicity Assay Using Ex Vivo γ-Oscillations

For electrophysiological experiments, all chemical compounds used in extracellular solutions were obtained from Sigma-Aldrich Sweden AB (Stockholm, Sweden). Kainic acid (KA) was obtained from Tocris Bioscience (Bristol, UK). WT Tau_441_ and the Tau_P301L_ mutant were expressed in with a NT* solubility tag that was cleaved out from the final Tau proteins and were purified with a Ni-NTA column and a Superdex 200 column, as described previously.^49^ Fibrils were prepared for both wild-type Tau_441_ and the Tau_P301L_ mutant in a buffer of NaP (pH 7.4), with 150 mM NaCl and 0.2 mM EDTA with agitation in the presence of heparin, and the fibril formation was confirmed by EM. To test the effect of acute exposure to WT Tau_441_ and Tau_P301L_ by incubation on ex vivo hippocampal γ-oscillations, we used WT mice (*N* = 14) at 4–6 postnatal weeks (ethical permit: 12570-2021 approved by the Stockholm Animal Ethical Board). The mice were deeply anesthetized with isoflurane before brain extraction. The brain was dissected out and placed in ice-cold artificial cerebrospinal fluid (ACSF) modified for dissection containing 80 mM NaCl, 24 mM NaHCO_3_, 25 mM glucose, 1.25 mM NaH_2_PO_4_, 1 mM ascorbic acid, 3 mM Na-pyruvate, 2.5 mM KCl, 4 mM MgCl_2_, 0.5 mM CaCl_2_, and 75 mM sucrose. The ACSF was bubbled with carbogen (95% O_2_ and 5% CO_2_). Horizontal sections (350 μm thick) of the ventral hippocampi of both hemispheres were prepared with a Leica VT1200S vibratome (Leica Microsystems). The slices, after cutting, were transferred into a humidified interface holding chamber containing standard ACSF (124 mM NaCl, 30 mM NaHCO_3_, 10 mM glucose, 1.25 mM NaH_2_PO_4_, 3.5 mM KCl, 1.5 mM MgCl_2_, and 1.5 mM CaCl_2_), continuously supplied with humidified carbogen. During slicing, the chamber was held at 37 °C and afterward allowed to cool down to room temperature (∼22 °C) for a minimum of 1 h. Six conditions to test the toxic effect of Tau and Bri2 BRICHOS monomers on hippocampal γ-oscillations are presented in the following order: (1) control group (20 mM NaP (pH 7.4), buffer, 150 mM NaCl, 0.2 mM EDTA), (2) 200 nM WT Tau_441_ monomers and (3) fibrils, (4) 200 nM Tau_P301L_ monomers and (5) fibrils, and (6) 200 nM Tau_P301L_ + 200 nM monomeric Bri2 BRICHOS. The protein samples were added to the hippocampal slices in 20 mM NaP (pH 7.4), buffer supplemented with 150 mM NaCl and 0.2 mM EDTA. Before extracellular recording measurements, the hippocampal slices were preincubated in each condition for 30 min in a submerged incubation chamber containing ACSF. The slices were supplied continuously with carbogen gas (5% CO_2_, 95% O_2_) bubbled into the ACSF during the incubation. After the preincubation, the slices were transferred to the interface-style recording chamber for the extracellular recording measurements.^35,55,56^

The extracellular recordings were measured with borosilicate glass microelectrodes filled with ACSF in the hippocampal area CA3, pulled to a resistance of 3–6 MΩ. An interface-type chamber (perfusion rate: 4.5 mL/min) was used to record local field potentials (LFP) at 32 °C. A 100 nM concentration of kainic acid was used to elicit the LFP γ-oscillations. Before the measurements were recorded, the oscillations were stabilized for 20 min. Interface chamber LFP recordings were carried out by a 4-channel amplifier/signal conditioner M102 amplifier (Electronics Lab, University of Cologne, Germany). The signals were sampled at 10 kHz, conditioned using a Hum Bug 50 Hz noise eliminator (Quest Scientific, North Vancouver, BC, Canada), software low-pass filtered at 1 kHz, digitized, and stored using Digidata 1322 A and Clampex 10.4 programs (Molecular Devices, CA, USA). Power spectra density plots from 60 s long LFP recordings were calculated in averaged Fourier segments of 8192 points using an Axograph X (Kagi, Berkeley, CA, USA). By integrating the power spectral density between 20 and 80 Hz, the γ-oscillations power was calculated, with the result representing average values for 1 min times.^57^

### Statistics

ThT fluorescence kinetics experiments were repeated at least three times with representative results and were performed in six replicates. Data are presented as medians with standard deviations. Turbidity measurements are presented as averages with standard deviation values from three replicates and repeated at least three times. The statistical significance for the toxicity assay using ex vivo γ-oscillations was estimated by one-way ANOVA, followed by Tukey’s multiple comparisons test (monomers) and uncorrected Fisher’s LSD for fibrils. For monomers, *F* = 1.492 with DF 4, and for fibrils, *F* = 3.350 with DF 3. Results are presented as the mean ± SEM. Statistical analyses were performed using GraphPad Prism (GraphPad, La Jolla, CA). The *p*-values used were *p < 0.05, **p < 0.01, and nonsignificant (ns) values *p* > 0.05. To calculate the statistics, the following *N* values for the six conditions were used: (1) control group *N* = 13 (monomers) and *N* = 15 (fibrils), (2) 200 nM WT Tau_441_ monomers *N* = 9 and (3) fibrils *N* = 8, (4) 200 nM Tau_P301L_ monomers *N* = 9 and (5) fibrils *N* = 13, and (6) 200 nM Tau_P301L_ + 200 nM monomeric Bri2 BRICHOS *N* = 13.

## Results

### Bri2 BRICHOS Inhibits Tau_441_ Aggregation by Predominately Preventing Secondary Nucleation Processes

To follow the nucleation-dependent conversion from a soluble, monomeric state to an insoluble fibrillar state, we applied aggregation kinetics assays. Bri2 BRICHOS populates differently sized assembly states with distinct features in client specificity.^28,36^ Well-characterized fractions of both monomeric and oligomeric Bri2 BRICHOS were isolated and collected by using gel filtration. The aggregation kinetics experiments were performed with cofactor-free full-length Tau_441_, applying continuous agitation with glass beads in the presence of different concentrations of monomeric and oligomeric Bri2 BRICHOS. We observed that Tau_441_ protein fibrillation is delayed by both oligomeric (Figure 1B) and monomeric (Figure S2) Bri2 BRICHOS in a concentration-dependent manner, with a strong inhibitory effect already at a low molar ratio of Bri2 BRICHOS/Tau_441_. The aggregation halftime, *t*_1/2_, describing the time when half of the Tau_441_ monomers are depleted, increases from ∼30 to 90 h for 20 μM Tau_441_ in the presence of 3% molar equivalent of oligomeric Bri2 BRICHOS (Figure 1B). The effect is similar, but less efficient, for 3% monomeric Bri2 BRICHOS with an increase of *t*_1/2_ value to ∼40 h (Figure S2). Notably, at only 5% molar equivalent of either oligomeric or monomeric Bri2 BRICHOS, the fibrillation process of Tau_441_ is basically completely inhibited during the tested time course (Figures 1B and S2).

Due to the stronger effect of oligomeric Bri2 BRICHOS, this species is primarily studied in this report with complementary data for monomeric Bri2 BRICHOS in Supporting Information.

To obtain information about the underlying microscopic nucleation processes from the bulk aggregation kinetics profiles, we applied a global fit analysis of the kinetic traces using different nucleation models.^51^ We found that a secondary nucleation-dominated model, including the microscopic nucleation rates of primary nucleation, *k*_*n*_, fibril-end elongation, *k*_*+*_, and surface-catalyzed secondary nucleation, *k*_2_, fits well to our data (Figure 1B). We further investigated the effect of the individual nucleation rates by setting one nucleation rate as a free individual fitting parameter and constraining the other two as global fitting parameters. Following this approach, we found that the fits for the elongation rate, *k*_*+*_, and/or the secondary nucleation, *k*_2_, as free fitting parameters best describe the kinetic traces, indicating that the inhibitory effect mainly originates from a reduction of *k*_*+*_ and/or *k*_2_ (Figure 1B). To confirm this observation, we performed aggregation kinetics experiments with 1% preformed Tau_441_ seeds, where the presence of seeds basically abolishes the contribution of *k*_*n*_. Indeed, we found that the inhibitory effect of Bri2 BRICHOS is still evident, and the aggregation traces fit well to a secondary nucleation-dominated model where an additional parameter, *M*_0_, for the initial seed concentration is included, and *k*_*n*_ is held constant, giving further support that primary nucleation is not affected by the presence of Bri2 BRICHOS (Figure S3). To differentiate whether *k*_*+*_ or *k*_2_ is the most affected nucleation rate, we conducted highly seeded kinetics with 20% preformed Tau_441_ seeds (Figure S3). Under these conditions, numerous fibrillar ends are available, resulting in fibril-end elongation events dominating the early time points of the aggregation reaction. Remarkably, even in the presence of 20% seeds, the aggregation trace exhibited a curve shape with a lag time and an exponential phase. In contrast, 3% Bri2 BRICHOS drastically inhibits 20% seeded Tau_441_ fibrillation, resulting in an almost flat kinetic trace. The observed curve shapes make it difficult to accurately determine the slope of the aggregation trace during the first time points (typically concave curve shapes are used for this kind of analysis), resulting in us not distinguishing the respective effect on *k*_*+*_ and *k*_2_. Hence, we conclude that secondary nucleation processes, involving *k*_*+*_ and *k*_2_, are affected by Bri2 BRICHOS.

### Bri2 BRICHOS Interacts with Tau_441_ Monomers

To elucidate the molecular basis of the observed inhibitory effect of Bri2 BRICHOS in detail, we investigated the interactions of Bri2 BRICHOS with monomeric Tau_441_. Dynamic light scattering (DLS) experiments were conducted to detect the hydrodynamic radii of the proteins alone and as a mixture (Figure 2A). Monomeric Tau_441_ showed one well-dispersed peak with a hydrodynamic radius of 5.7 nm, and oligomeric Bri2 BRICHOS alone exhibits a peak of 11.5 nm. In contrast, for the mixed sample of oligomeric Bri2 BRICHOS with Tau_441_, a shift of the peaks to 17.5 nm was observed, indicating a larger complex. To further explore this finding, Bri2 BRICHOS samples with and without Tau_441_ monomers were mixed and followed by native-PAGE analysis (Figure S4). Oligomeric Bri2 BRICHOS exhibits one broad peak in DLS experiments but shows multiple bands on a gel during native conditions. Remarkably, in the presence of Tau_441_ monomers, the bands of Bri2 BRICHOS weakened by at least 50%, indicating an interaction of Bri2 BRICHOS and Tau_441_ monomers forming larger protein assemblies and complexes.

**Figure 2:**
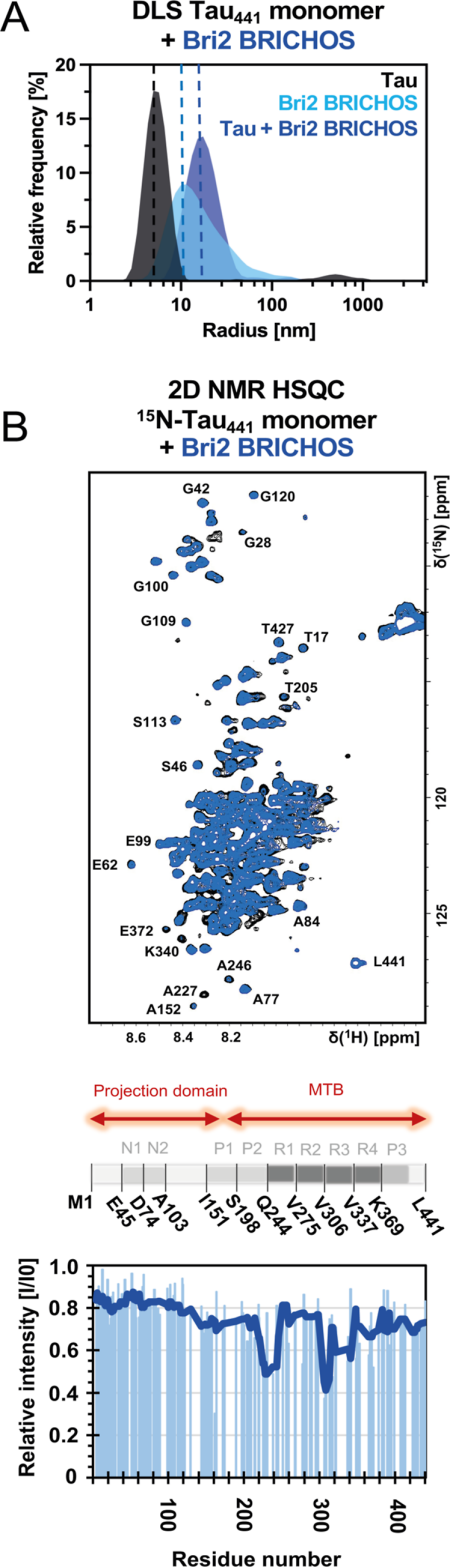
Bri2 BRICHOS interacts with Tau_441_ monomers. (A) DLS profiles of 25 μM Tau_441_ and 12.5 μM oligomeric Bri2 BRICHOS, suggesting the formation of larger complexes for the Tau-BRICHOS mixture. (B) ^1^H–^15^N-HSQC NMR spectrum of 100 μM ^15^N-labeled monomeric Tau_441_ (black) in the presence of 100 μM oligomeric Bri2 BRICHOS (blue). The NMR experiments were performed at 298 K in 20 mM NaP buffer, pH 7.4. Residues with a too low signal-to-noise ratio (<12) or overlap were excluded from the analysis. The solid line represents a smoothing function of 15 using the median. MTB refers to the microtubule-binding domain, and I/I0 is the relative NMR signal intensity of ^15^N-Tau_441_ with and without Bri2 BRICHOS.

To shed more light on the Bri2 BRICHOS-Tau_441_ monomer interactions, we sought to obtain high-resolution details by applying solution NMR spectroscopy. ^1^H–^15^N-HSQC experiments using 100 μM ^15^N-labeled monomeric Tau_441_ were recorded in the presence of Bri2 BRICHOS, allowing us to follow the signal intensity and chemical shift changes (Figures 2B and S4). The addition of substoichiometric to equimolar concentrations of Bri2 BRICHOS resulted in an attenuation of certain cross-peak signal intensities with no or only minor chemical shift changes, whereas other cross-peaks were not affected. The presence of Bri2 BRICHOS caused an overall signal attenuation, where specific regions were more affected. In particular, residues 220–240 and 300–320, comprising the microtubule binding domain region, exhibited a significant averaged signal reduction compared to the less affected N- and C-terminal regions. The lack of any significant chemical shift changes suggests no major structural changes upon the interaction. Circular dichroism (CD) spectra did not show any changes, supporting this conclusion (Figure S5). Complementarily, we used ^15^N-labeled monomeric Bri2 BRICHOS (as the size of oligomeric Bri2 BRICHOS causes significant line broadening) to monitor the interaction with unlabeled monomeric Tau_441_ (Figure S4). Notably, due to the dynamic nature of Bri2 BRICHOS, only the N-terminal residues are visible in the ^1^H–^15^N-HSQC spectrum.^31^ We found that the signal intensities of all visible cross-peak intensities for 68 μM ^15^N-labeled Bri2 BRICHOS in the presence of 34 μM monomeric Tau_441_ were clearly reduced by 40–60%, in particular in the region from residues 6 to 25 corresponding to β-strand regions in Bri2 BRICHOS. Even though an interaction between Bri2 BRICHOS and monomeric Tau_441_ is evident, a well-defined binding site in the NMR-visible region is not observed.

Based on these results, we conclude that Bri2 BRICHOS dynamically binds to monomeric Tau_441_ and interferes with the monomer-dependent elongation and secondary nucleation processes of Tau_441_ fibrillation.

### Bri2 BRICHOS Regulates Tau_441_ Condensate Formation

Tau_441_ forms biomolecular condensates, droplets, in buffer solutions via LLPS processes,^3^ and this process can be induced by molecular crowding agents,^10^ like PEG8000, which we applied here. It has been demonstrated that PEG is excluded from the droplets (the dense phase).^14^ Under our experimental conditions, Tau_441_ instantly formed droplets in the presence of 8% PEG8000 with a diameter of up to a few micrometers (Figure 3A). The droplets exhibited liquid-like behavior with fusion and growth over time. On the contrary, Bri2 BRICHOS did not form any droplets (Figure 3B). In the presence of Bri2 BRICHOS at substoichiometric concentrations, we found only minor effects on the Tau_441_ droplets (Figure 3A). A more pronounced effect was observed at a superstoichiometric concentration of Bri2 BRICHOS, where no droplets were visible. Also, when Bri2 BRICHOS was supplemented at superstoichiometric concentrations to already formed and stable Tau_441_ droplets, after 1 day of incubation, we observed a dissolution of the droplets (Figure S6). To validate the findings of modulated Tau_441_ LLPS behavior, we followed the phase separation by measuring the turbidity, where increased turbidity values indicate droplet formation. Again, substoichiometric concentrations of Bri2 BRICHOS showed no increase in turbidity, whereas an equimolar concentration resulted in a 2-fold increased absorbance value compared to Tau_441_ droplets alone (Figure 3C), supporting the findings by microscopy images (Figure 3A). Notably, slightly higher turbidity values were measured when Bri2 BRICHOS was added to a mixture of Tau_441_ and PEG8000, compared to when PEG8000 was added directly to a Tau_441_ and Bri2 BRICHOS mixture (Figure S7). However, no observable changes in the appearance of the droplets were observed (Figure S7). The turbidity was also followed as a function of PEG8000 concentration, where Bri2 BRICHOS enhanced the droplet formation, as evident by an increased turbidity at lower PEG8000 concentrations (Figure S7).

**Figure 3:**
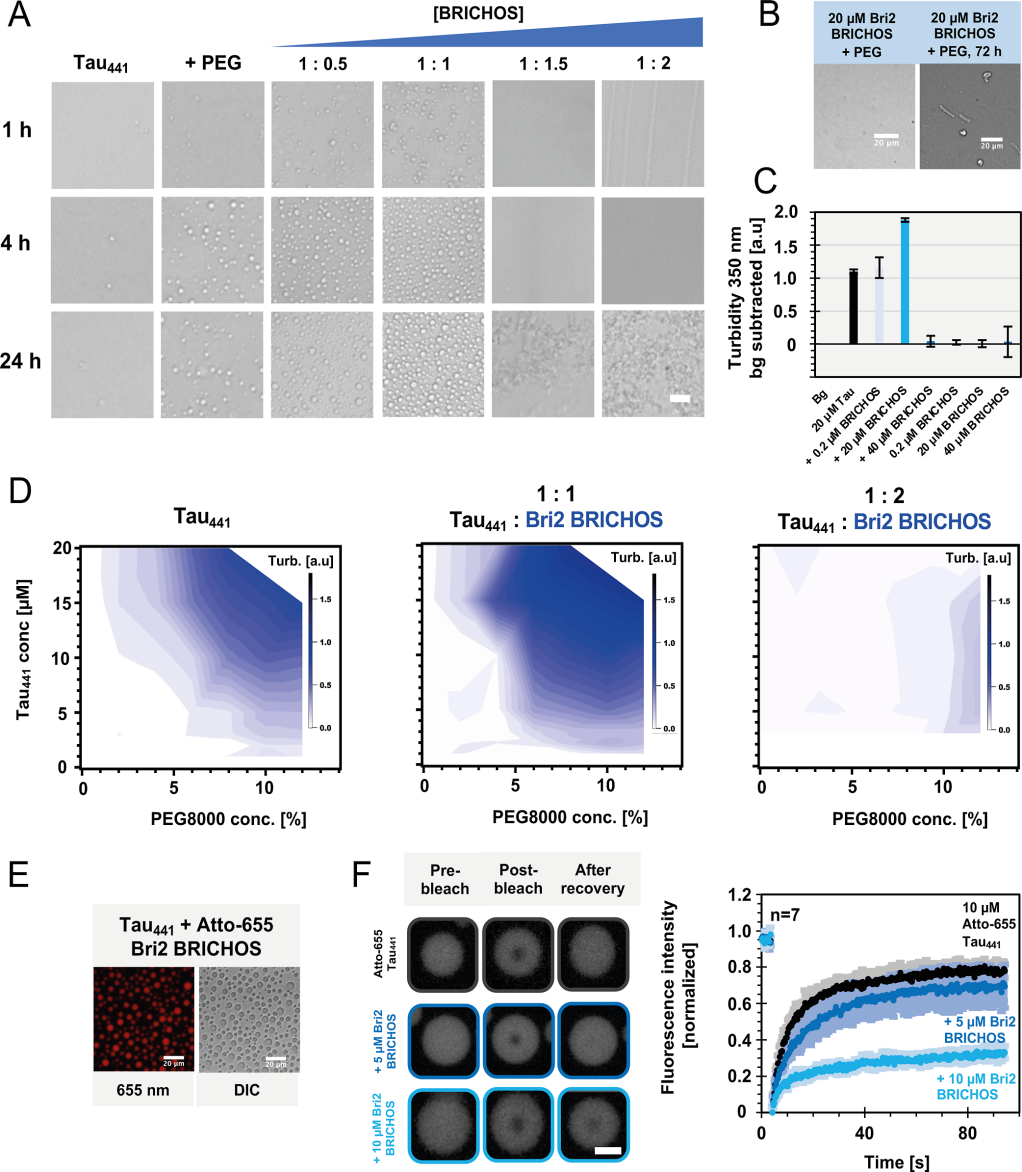
Bri2 BRICHOS regulates Tau_441_ condensate formation. (A) Brightfield microscopy images of 10 μM Tau_441_ in 8% PEG8000, 20 mM NaP, pH 7.4, at room temperature, in the presence of different concentrations of oligomeric Bri2 BRICHOS, at three different time points. Droplet formation is promoted in the presence of a 1:1 ratio but completely abolished above equimolar Bri2 BRICHOS concentrations. (B) Bri2 BRICHOS alone does not form droplets during the experimental time. (C) Turbidity (absorbance at 350 nm) values of freshly prepared 20 μM Tau_441_ in the presence of Bri2 BRICHOS, showing high values for Tau_441_ alone and at sub- and equimolar concentrations of Bri2 BRICHOS, which is linked to droplet formation, and low values for superstoichiometric ratios of Bri2 BRICHOS and Bri2 BRICHOS alone. Average and standard deviation values from *N* = 3 replicates are shown, where the error bars represent the standard derivation. (D) Phase diagrams of turbidity values at varying Tau_441_ and PEG8000 concentrations in the absence and presence of oligomeric Bri2 BRICHOS. (E) Fluorescence and DIC images of 20 μM Tau_441_ in the presence of 1% monomeric Bri2 BRICHOS fluorescently labeled with Atto-655 dye, showing that Tau_441_ forms droplets and that Bri2 BRICHOS is incorporated into the Tau_441_ droplets. (F) FRAP experiments with 10 μM Tau_441_ (1% fluorescently labeled with Atto-655), 8% PEG8000, and 5 or 10 μM oligomeric Bri2 BRICHOS, demonstrating that the protein dynamics within the droplets is attenuated in the presence of Bri2 BRICHOS. The scale bar is 20 μm in (A) and 2.5 μm in (F).

We continued to systematically investigate the effect of Tau_441_ and PEG8000 concentrations, resulting in phase diagrams (Figure 3D). Comparing the phase diagrams of Tau_441_ alone and in the presence of equimolar concentrations of Bri2 BRICHOS, we found increased turbidity values related to an increased droplet propensity, or alternatively, Bri2 BRICHOS incorporation causes the increased turbidity values. In contrast, basically no increased turbidity values were measured at superstoichiometric Bri2 BRICHOS concentrations, indicating that no phase separation occurs under these conditions. Interestingly, after 24 h, Bri2 BRICHOS alone still displayed low turbidity values, but for Tau_441_ in the presence of superstoichiometric concentrations of Bri2 BRICHOS, highly increased turbidity values were detected (Figure S8). This observation suggests that the system matured without the formation of fibrils or droplets. To follow the maturation of Tau_441_ droplets over time, we monitored the droplets with and without low concentrations of Bri2 BRICHOS after 100 h (Figure S7). We found that several Tau_441_ droplets exhibited changes in shape and structure, whereas the droplets with Bri2 BRICHOS did not, suggesting that Bri2 BRICHOS can interfere with the maturation of Tau_441_ droplets.

To further elucidate how Bri2 BRICHOS interacts with Tau_441_ droplets, we fluorescently labeled Bri2 BRICHOS (1%) and found that Bri2 BRICHOS is compartmentalized within Tau_441_ droplets (Figure 3E). *Z*-stacking of single imaging plans reveals a uniform distribution of Bri2 BRICHOS within droplets (Figure S9). The incorporation was stable for at least 72 h, where the droplets were able to fuse and form larger condensates. As a confirmation of the specific interaction of Tau_441_ and Bri2 BRICHOS, we repeated the same type of experiment with a control protein, possessing a similar size and pI as Bri2 BRICHOS, for which we used NT*,^54^ where no change in droplet formation nor incorporation into the droplets was found (Figure S10).

The incorporation of Bri2 BRICHOS raises the question of whether Bri2 BRICHOS modifies the intrinsic Tau_441_ droplet properties. To elucidate if Bri2 BRICHOS interferes with the typical Tau_441_ liquid-like properties and intradroplet diffusion, fluorescence recovery after photobleaching (FRAP) experiments with fluorescently labeled Tau_441_ were conducted (Figure 3F). Fresh Tau_441_ droplets exhibited a nearly full recovery of the fluorescence signal after 100 s, as previously described in the literature.^11^ The liquid-like behavior of Tau_441_ droplets at a 1:0.5 molar ratio of Tau_441_/Bri2 BRICHOS was basically not affected. In contrast, at equimolar concentrations, the fluorescence signal recovered only to about 30% of the initial intensity, indicating a slower Tau_441_ diffusion within the droplets (Figure 3F). Together, our results suggest that Bri2 BRICHOS is recruited into Tau_441_ droplets and increases the turbidity of Tau_441_ droplets (Figure 3C,E), facilitating an environment where Tau_441_ molecules are dynamically restricted (Figure 3F).

### Molecular Insights into the Modulation of Tau_441_ Droplet Formation by Bri2 BRICHOS

To obtain mechanistic insights into the modulating behavior of Bri2 BRICHOS on Tau_441_ condensation, we further investigated the Tau_441_ and Bri2 BRICHOS interactions in a droplet state by CD and NMR spectroscopy. We observed that the CD spectra of 5 μM Tau_441_ with and without 10 μM Bri2 BRICHOS in the presence of PEG8000 basically remained the same as the spectra in the absence of the molecular crowding agent, suggesting no significant changes of the secondary structures (Figure S11). We further conducted 2D NMR ^1^H–^15^N-HSQC experiments of 62 μM ^15^N-labeled Tau_441_ with and without 6% PEG8000 and compared the cross-peak intensities and chemical shift differences (Figure 4). In the presence of PEG8000, most of the spectrum remained the same as in the absence of PEG8000, but with minor chemical shift changes and a small reduction in the signal intensities. The effect of PEG is in line with previously reported data, where a slower tumbling rate and exchange with an invisible droplet state were suggested to explain the intensity attenuation.^60,61^ Of note, 80–90% of the overall signal intensity remained, related to visible Tau_441_ resonances under droplet-state conditions. Next, we added equimolar concentrations of Bri2 BRICHOS (Figures 4A and S12) to Tau_441_ in the presence of PEG8000. The presence of Bri2 BRICHOS caused a decrease in signal intensities of several cross-peaks as well as chemical shift changes (Figures 4B–D and S12). Remarkably, the induced chemical shift changes of Bri2 BRICHOS added to Tau_441_ droplets overall reverse the PEG8000-induced chemical shift changes of Tau_441_, with many cross-peaks shifting back to the values without PEG8000 (Figure 4B,D). A possible explanation for this observation is that the interaction between Tau_441_ and Bri2 BRICHOS counteracts the multivalent interactions responsible for the droplets. Further, the relative intensities (Figure 4C) were compared with previous data without PEG8000 (Figure 2B), where an overall similar pattern of the NMR signal intensities was observed but with stronger effects on residues 140–200 and the C-terminus, suggesting similar interactions between Tau_441_ and Bri2 BRICHOS both in homogeneous solution and under droplet conditions.

**Figure 4:**
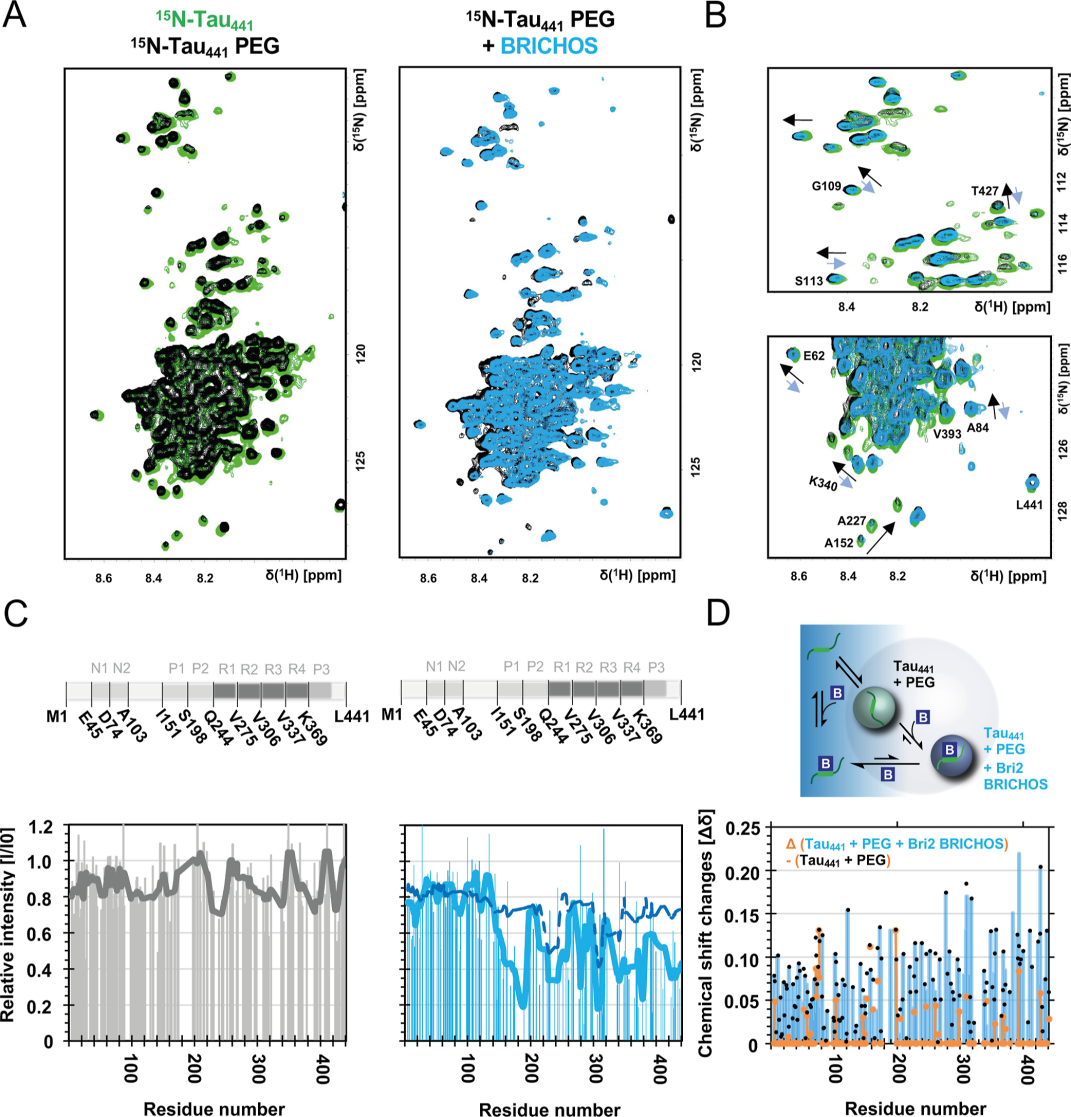
Bri2 BRICHOS interacts with Tau_441_ monomers in the droplet state. (A,B) 2D NMR ^1^H–^15^N-HSQC experiments with 62 μM ^15^N-labeled monomeric Tau_441_ (green) in the presence of 6% PEG8000 (black) and upon addition of 62 μM oligomeric Bri2 BRICHOS (blue). The experiments were conducted at 298 K in 20 mM NaP buffer, pH 7.4. (C) The signal intensity changes upon addition of PEG and oligomeric Bri2 BRICHOS. The solid lines represent smoothing functions over 15 data points using the median. The dashed line corresponds to data without PEG for ^15^N-Tau_441_ and Bri2 BRICHOS from Figure 2. (D) Bri2 BRICHOS (denoted B in the figure) binds to Tau (denoted as a green monomer) both in buffer and in droplets and shifts the equilibrium of Bri2 BRICHOS-Tau droplet complexes to soluble complexes in the dilute phase. Chemical shift changes for Tau_441_ and PEG8000 (black dots) and Tau_441_, PEG8000, and Bri2 BRICHOS (blue bars) and the difference (orange), showing that the presence of Bri2 BRICHOS (blue bars) counteracts the induced chemical shift changes by PEG8000 (black dots) for most residues. Residues with a too low signal-to-noise ratio (<12) or overlap were excluded from the analysis.

### Bri2 BRICHOS Inhibits Tau_P301L_ Fibril-Induced Toxicity on the Hippocampal Network Activity

Hippocampal γ-oscillations are associated with higher cognitive functions, such as memory formation and learning processes.^62^ Prior research has highlighted the role of γ-oscillation reduction in various pathological conditions characterized by cognitive impairment, including AD^63^ and other tauopathies.^64^ To follow whether Bri2 BRICHOS interferes with Tau-induced adverse effects on the hippocampal network activity, we engaged an ex vivo model system investigating the impact on cognition-relevant γ-oscillations in mouse hippocampal brain slices. Wild-type Tau_441_ (200 nM) and the disease-related P301L mutant Tau_P301L_ (200 nM) were applied to the hippocampal slices, either in a monomeric or fibrillar state, with and without the presence of 200 nM monomeric Bri2 BRICHOS. Neither Tau_441_ nor Tau_P301L_, in a monomeric state at concentrations below physiological concentrations, exhibited any statistically significant neurotoxicity, as observed by the lack of a statistically significant decrease in the power of the γ-oscillations (Figures 5 and S13). Interestingly, while fibrillar WT Tau_441_ did not show any significant effect, fibrillar Tau_P301L_ induced a significant decrease (*p* = 0.005) in the power of the γ-oscillations. In the presence of monomeric Bri2 BRICHOS, this effect was abolished (*p* = 0.47 to control and *p* = 0.045 to fibrillar Tau_P301L_). Notably, Tau_P301L_ is much more aggregation-prone than WT Tau_441_, and Bri2 BRICHOS also efficiently delays Tau_P301L_ fibrillation and modulates its droplet formation similarly to WT Tau_441_ (Figure S14). Hence, these observations indicate that Bri2 BRICHOS can suppress Tau-associated toxicity, in particular, of the more toxic Tau_P301L_ variant, in a hippocampal slice preparation toxicity model system.

**Figure 5:**
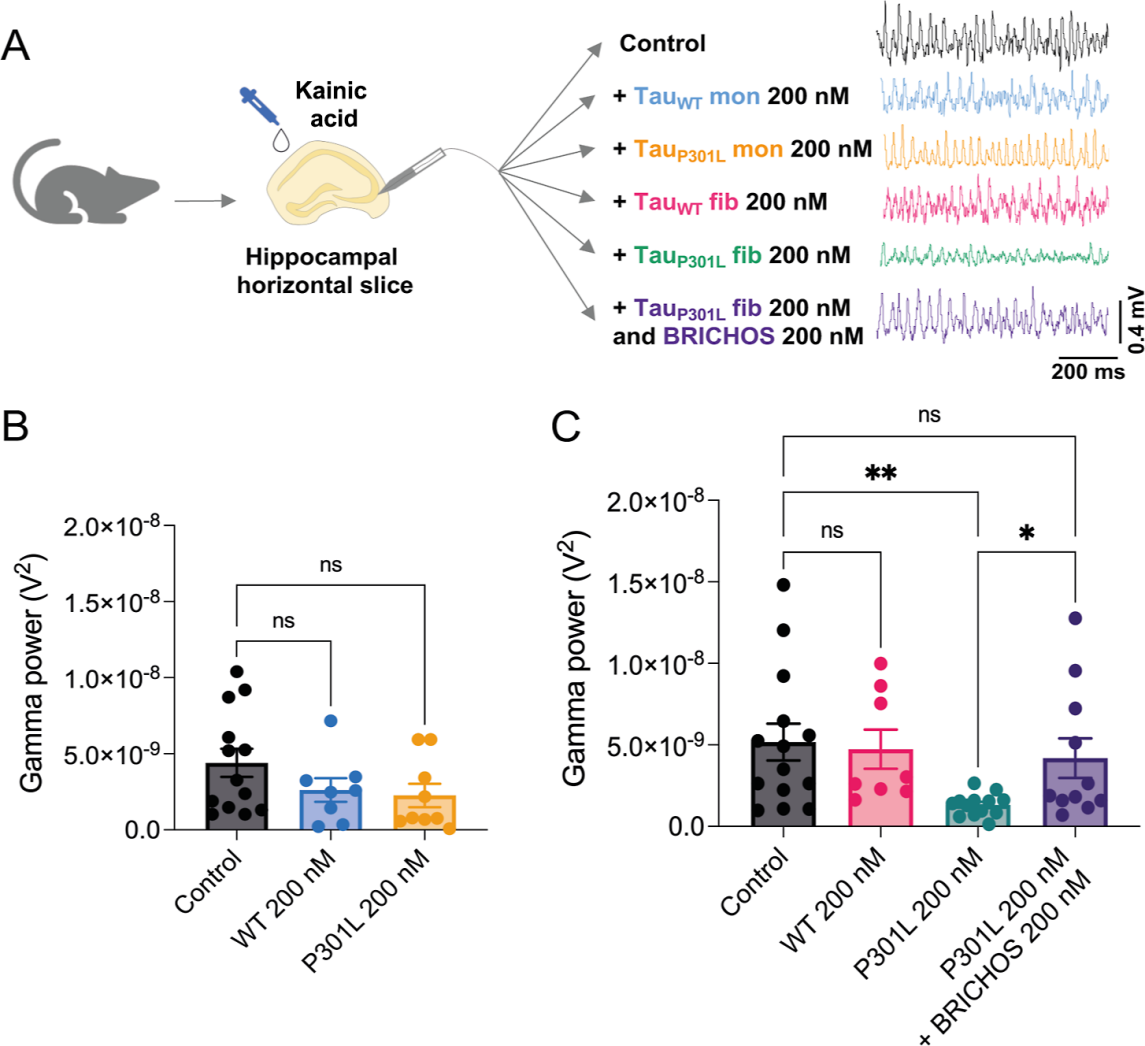
Bri2 BRICHOS inhibits toxicity associated with fibrillar Tau_P301L_. (A) Experimental setup of the ex vivo model system measuring the impact on γ-oscillations in mouse hippocampal brain slices with representative electrophysiology traces for each condition. (B) γ-Oscillation power in the presence or absence of 200 nM WT Tau_441_ monomers (blue) or Tau_P301L_ monomers (yellow), revealing no significant impact. (C) γ-Oscillation power in the presence or absence of 200 nM WT Tau_441_ fibrils (pink), Tau_P301L_ fibrils (green), or Tau_P301L_ fibrils coincubated with 200 nM monomeric Bri2 BRICHOS (purple), showing significantly reduced γ-oscillation power by fibrillar Tau_P301L_, which can be prevented in the presence of monomeric Bri2 BRICHOS. The data in (B,C) are expressed as the mean ± SEM, and the statistical significance was estimated by one-way ANOVA, followed by Tukey’s multiple comparisons test for monomers and uncorrected Fisher’s LSD for fibrils. *p < 0.05, **p < 0.01, ns, nonsignificant.

## Discussion

Tau_441_ can form both liquid droplets and amyloid fibrils. The present research provides insights into how a molecular chaperone can be used to efficiently target such harmful processes and suggests that the regulation of droplet formation can potentially modulate Tau aggregation and subsequent toxicity.

The current results show binding of Bri2 BRICHOS to both monomeric and fibrillar Tau_441_, resulting in the inhibition of Tau fibrillation by interference with secondary nucleation processes (Figures 1 and 6). Inhibition of secondary nucleation pathways by BRICHOS has already previously been observed for Aβ, IAPP, and α-synuclein.^28,30,31,65^ Of note, the presence of fragmentation processes (referred to as monomer-independent secondary nucleation) in the overall fibrillation process cannot be excluded, especially under experimental conditions where agitation is applied. However, there is no reasonable model for the inhibitory effect of Bri2 BRICHOS on fibril fragmentation. Like Tau, α-synuclein also exhibits fibrillation kinetics with possible contribution of both surface-catalyzed secondary nucleation and fragmentation processes, but detailed studies indicate surface-catalyzed secondary nucleation as the dominant fibrillation mechanism,^31,66^ suggesting that a similar mechanism of action may also be present for Bri2 BRICHOS inhibition of Tau_441_ aggregation. In contrast to Tau_441_ (Figure 2), Bri2 BRICHOS does not bind to either monomeric Aβ or α-synuclein.^28,31,65^ These polypeptides are strikingly smaller compared to full-length Tau_441_ and they are, like Bri2 BRICHOS, overall negatively charged as opposed to the positively charged Tau_441_. On the contrary, Bri2 BRICHOS binds to the fibrillar states of all these peptides and proteins, which is intimately connected to the inhibitory effect of secondary nucleation reactions.^32,67,68^ While for Tau, future experiments may reveal the specific binding site of Bri2 BRICHOS on the fibril surface, a recent study showed that Bri2 BRICHOS recognizes the three C-terminal β-strands of Aβ_42_ fibrils.^32^ Thus, the generic ability of Bri2 BRICHOS to bind these and other fibrils^67^ might originate in its ability to bind β-structures.

**Figure 6:**
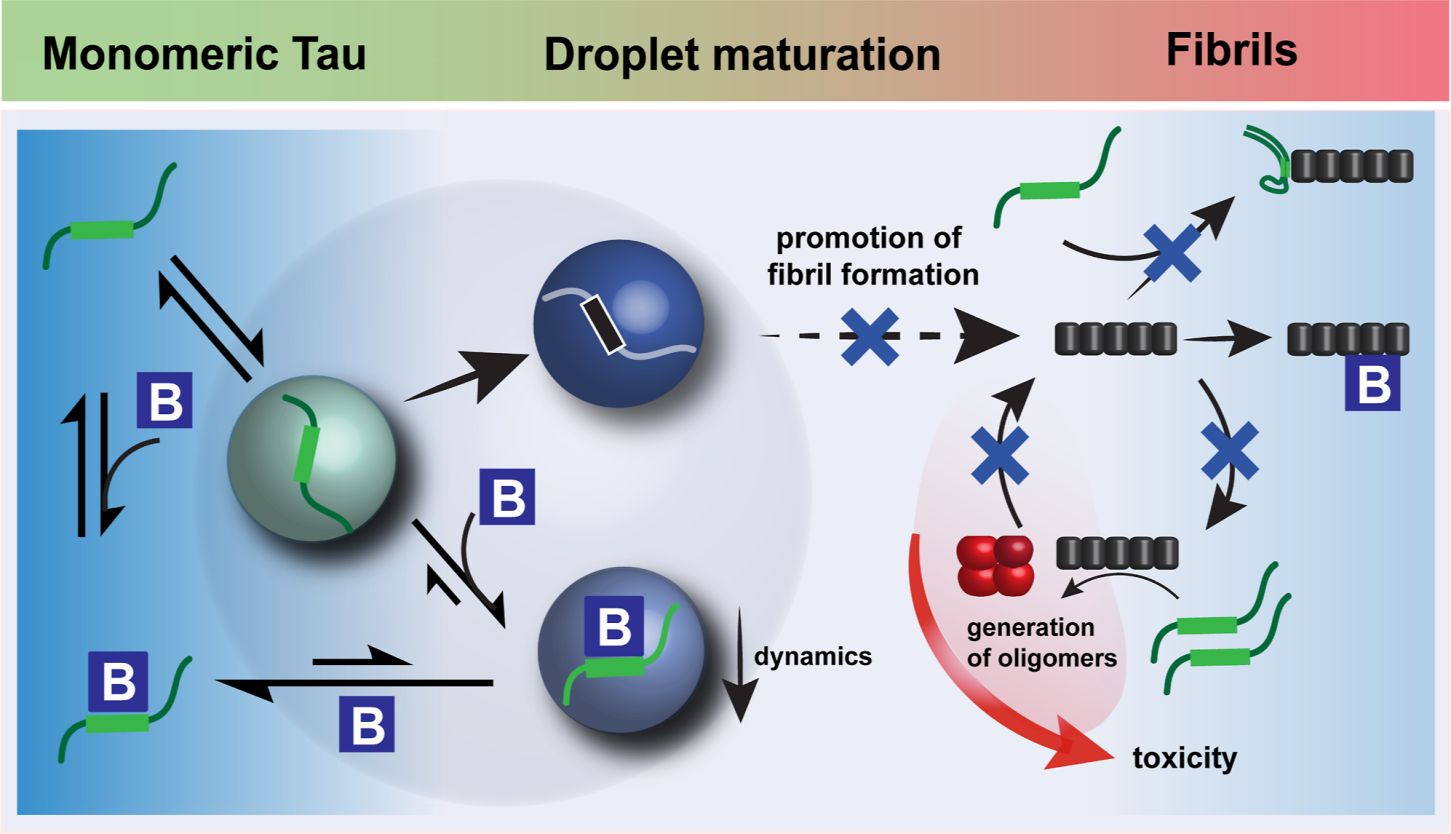
Mechanism of action of regulation of Tau_441_ phase separation and aggregation by Bri2 BRICHOS. Intrinsically disordered, monomeric Tau_441_ forms liquid-like droplets via LLPS processes. Bri2 BRICHOS modulates these processes by interactions with Tau_441_ both in solution and in the droplet state, where the dynamics of Tau_441_ are attenuated and a structural state of Tau_441_ that is less prone to droplet maturation is promoted by Bri2 BRICHOS. At a high molar ratio of Bri2 BRICHOS/Tau_441_, droplet formation is completely prevented. Maturation of the droplet state, which is counteracted by Bri2 BRICHOS, might represent an early precursor of Tau_441_ aggregation. Additionally, Bri2 BRICHOS binding to Tau_441_ monomers and fibrils predominately inhibits secondary nucleation—a process that is potentially connected to the generation of (neurotoxic) oligomers. Targeting these processes may hence be linked to the observed suppression of Tau-induced toxicity by Bri2 BRICHOS.

The interactions between monomeric Tau_441_ and Bri2 BRICHOS are mainly localized to the aggregation-prone regions in the Tau_441_ sequence (Figure 2). Remarkably, these interactions occur both in aqueous solution (Figure 2) and in the droplet state (Figure 3). At low Bri2 BRICHOS concentrations ([Bri2 BRICHOS]​ ≪ [Tau]), Bri2 BRICHOS is integrated inside the Tau droplets without any detectable changes in the droplet size, yet preventing maturation of Tau_441_ droplets (Figures 3E and S7). At equimolar protein concentrations, the incorporation of Bri2 BRICHOS in the Tau_441_ droplets gives rise to increased turbidity values (Figure 3C), together with a decrease in the Tau dynamics and fluidity as measured by FRAP experiments (Figure 3F). During these conditions, Bri2 BRICHOS interactions with Tau_441_ seem to enhance phase separation. At a 2:1 Bri2 BRICHOS/Tau_441_ molar equivalent, Bri2 BRICHOS completely prevents Tau droplet formation (Figure 3), potentially by energetically more favorable interactions between the Bri2 BRICHOS and Tau_441_ compared to Tau–Tau interactions within the droplet state, thereby dissolving the droplet structures. In contrast, for complete fibrillation inhibition, only a few percent of Bri2 BRICHOS relative to Tau_441_ are required (Figure 1B). While molecular interactions between Tau monomers within the PEG-induced droplets are modulated by Bri2 BRICHOS, possibly with both electrostatic and specific binding events, the drastic inhibitory effect on Tau fibrillation stems both from BRICHOS binding to Tau fibrils, which potentially blocks catalytic surfaces for secondary nucleation processes, and binding to Tau monomers. The versatile nature of Bri2 BRICHOS effects may originate from both electrostatics and more specific factors such as hydrophobic interactions and structural modulation of Tau–Tau interactions. Usage of the 14 kDa large control protein NT*, with a pI of 4.3 corresponding to a negative net charge at physiological pH, which is similar to Bri2 BRICHOS with a molecular weight of 14 kDa and a pI of 4.6, the same effects were not observed, suggesting specific Bri2 BRICHOS interactions rather than purely electrostatic interactions (Figure S10). Monomer-dependent secondary pathways have been shown to dominate Tau aggregation,^69,70^ and a perturbation of these nucleation reactions, both at the monomeric and fibrillar states of Tau, may therefore strongly modulate the aggregation behavior.

Upon addition of the molecular crowding agent PEG8000, which mimics excluded volume effects and wetting properties of a cellular environment,^71^ we observed chemical shift changes in the ^1^H–^15^N HSQC NMR spectrum of Tau (Figure 4B). Surprisingly, Bri2 BRICHOS was able to counteract these changes, giving rise to a spectrum more closely resembling Tau in the dilute phase. The Bri2 BRICHOS interactions with Tau droplets exhibit a less dynamic state (Figure 3F), suggesting changes in the properties of Tau in the droplet state that might be linked to a less aggregation-prone state.

In systems whose overall aggregation mechanism is dominated by fibril surface catalyzed secondary nucleation processes, like Tau,^69^ secondary nucleation is the major source for the generation of potentially neurotoxic oligomers.^68,72^ Our current results showed that Tau fibril-induced toxicity on the hippocampal network synchrony was prevented by Bri2 BRICHOS (Figure 5), suggesting a link between the inhibition and regulation effect of Tau aggregation and Tau droplet formation with decreased toxicity. The Bri2 BRICHOS properties of targeting Tau_441_ misfolded species and stabilizing nonaggregating Tau conformations further suggest that Tau droplet formation and aggregation are connected, in line with recent reports that droplets are formed during cofactor-free Tau aggregation.^47,73^ We rationalized our findings in a model representing the mechanism of action of Bri2 BRICHOS modulating Tau aggregation and LLPS processes and eventually Tau-induced toxicity (Figure 6).

Reviewing literature, some of the inhibitory effects reported here from Bri2 BRICHOS on Tau aggregation are shared with other molecular chaperones. Tau aggregates are clients to molecular chaperones studied both in vitro and in vivo, such as Hsp90,^74^ Hsp70,^20,75^ Hsp70/J-domain protein (JDP) families JDP DnaJC7,^76^ calcium-dependent S100B,^22^ Hsp22,^23^ cyclophilins,^77^ and clusterin.^78^ A common nominator seems to be binding of Tau and subsequent inhibition of the fibrillation kinetics, sharing properties with the current BRICHOS findings. Accumulating evidence suggests an inevitable role of molecular chaperones like heat shock proteins in LLPS processes.^23^ Like Bri2 BRICHOS, the proline-targeting protein peptidyl prolyl isomerase A (PPIA) is recruited into Tau droplets, and eventually, with an increased equimolar PPIA concentration, the droplets dissolve.^24^ Further, PPIA also binds to monomeric Tau_441_ with several affected regions.^24^ Another example is the protein disulfide isomerase (PDI), where Tau droplet formation and fibril formation were suppressed with reduced cytotoxicity.^79^ Unlike PDI^79^ and Bri2 BRICHOS, the calcium-dependent chaperone S100B did not induce any changes in the dynamics or fluidity of Tau droplets.^21^ Nevertheless, S100B still contributed to modulated and reduced droplet propensities and prevention of Tau fibrillation,^22^ suggesting similar effects as with Bri2 BRICHOS. In addition to molecular chaperones, small molecules like the anionic drug suramin or methylene blue were reported to possess dual effects to some extent, targeting both droplet formation and fibrillation of Tau.^61,80^

Most of the reported molecular chaperones are multimeric structures and are significantly larger than Bri2 BRICHOS, and typically, the relation of the effects on droplet formation, protein aggregation, and associated toxicity remained unclear. Since Bri2 BRICHOS exhibits properties shared with those of other LLPS and fibrillation modulators, parts of our current findings may be transferable to other systems. The dual effect of Bri2 BRICHOS on Tau and Aβ, both implicated in AD, provides a tool to target several pathogenic proteins with the same molecular chaperone. Translation of our results to the in vivo situation is appealing, where Tau is mainly intracellular, but under pathological conditions spreads from cell to cell and has been found in the extracellular space.^81^ On the contrary, the BRICHOS domain is part of a pro-protein eventually proteolytically cleaved off to the extracellular matrix.^25^ Recent results indicate that Bri2 BRICHOS can also cross cellular barriers^37,40,82,83^ and return to the cellular interior with potential interactions with Tau droplets. Hence, the Bri2 BRICHOS–Tau interaction might possibly occur both inside and outside the cell.

## Conclusions

This project demonstrated molecular insights into the link between the regulation of Tau droplet formation, inhibition of Tau aggregation, and subsequent rescue of Tau-induced neurotoxicity by Bri2 BRICHOS via interference of secondary nucleation processes and stabilization of monomeric Tau. Overall, the findings may give insights into how molecular chaperones counteract amyloid formation and toxicity, likely important for the design and development of therapeutics against AD and other tauopathies.

## Supplementary Material



## Notes

Additional experimental details, supporting and complementary results including monomeric Bri2 BRICHOS in ThT assays, CD spectroscopy, NMR spectra, phase diagrams, turbidity data, microscopy imaging, droplet partitioning experiments with control protein, γ-oscillation power experiments, and Tau_P301L_ protein fibrillation and phase separation behavior (PDF)

## Abbreviations

LLPS: liquid–liquid phase separation
ThT: thioflavin T
FRAP: fluorescence recovery after photobleaching
NMR: nuclear magnetic resonance
