# Supplementary material for: Chaperone-Mediated Regulation of Tau Phase Separation, Fibrillation, and Toxicity
Source: J Am Chem Soc. 2025 Jun 30;147(27):23504–18. doi: 10.1021/jacs.5c01369 (PMC12257531; doi:10.1021/jacs.5c01369)
Supplement: Supplementary file 1 [file ja5c01369_si_001.pdf]

Supporting Information

Chaperone-Mediated Regulation of Tau Phase Separation, Fibrillation, and Toxicity

Cecilia Mörman[1,2*], Axel Leppert[3,4], Giusy Pizzirusso[5,6], Zihan Zheng[1,7], Xun Sun[2], Rakesh Kumar[1,8], Henrik Biverstål[1], Michael Landreh[3,4], Jan Johansson[1], Luis Enrique Arroyo-Garcia[5], Jinghui Luo[2], Gefei Chen[1,3*], and Axel Abelein[1*]

[1] Department of Medicine Huddinge, Karolinska Institutet, 141 52 Huddinge, Sweden
[2] Center for Life Sciences, Paul Scherrer Institute, 5232 Villigen, Switzerland
[3] Department of Cell and Molecular Biology, Uppsala University, 751 24 Uppsala, Sweden
[4] Department of Microbiology, Tumor and Cell Biology, Karolinska Institutet, 171 65 Solna, Sweden
[5] Department of Neurobiology, Care Sciences and Society, Division of Neurogeriatrics, Center for Alzheimer Research, Karolinska Institutet, 171 77 Solna, Sweden
[6] Department of Women's and Children's Health, Karolinska Institutet, 171 77 Solna, Sweden
[7] Department of Pharmacology, Xi'an Jiaotong University, 710061 Shaanxi, China
[8] Department of Neurobiology, Care Sciences and Society, Division of Clinical Geriatrics, Center for Alzheimer Research, Karolinska Institutet, 141 83 Huddinge, Sweden

[*] Corresponding authors

Corresponding Authors information
cecilia.morman@ki.se, gefei.chen@ki.se, axel.abelein@ki.se

Table of Content

***Supporting Figures*……………………………………………………………………………………**

Figure S1. *Quality control of recombinant Tau$_{441}$ protein.*

Figure S2. *Cofactor-free Tau$_{441}$ aggregation is inhibited by both monomeric and oligomeric BRICHOS by inhibiting Tau$_{441}$ secondary nucleation processes.*

Figure S3. *The Bri2 BRICHOS domain inhibits Tau$_{441}$ protein fibrillation both in the presence of 1% or 20% pre-formed seeds.*

Figure S4. *Monomeric Bri2 BRICHOS interacts with Tau$_{441}$ monomers.*

Figure S5. *Secondary structures of Tau$_{441}$ in the absence and presence of Bri2 BRICHOS using Circular Dichroism (CD) spectroscopy.*

Figure S6. *Bri2 BRICHOS dissolves pre-formed Tau$_{441}$ droplets after one day.*

Figure S7. *Tau$_{441}$ droplet formation using different Bri2 BRICHOS and PEG8000 concentrations and the time dependence.*

Figure S8. *Microscopy images and phase diagrams of measured turbidity values for different Bri2 BRICHOS or Tau$_{441}$ protein and PEG8000 concentrations at different time points.*

Figure S9. *Confocal microscopy Z-stack image of Tau$_{441}$ droplets with fluorescently labeled Bri2 BRICHOS.*

Figure S10. *NT* (control protein) is not incorporated into the Tau$_{441}$ droplets.*

Figure S11. *Secondary structures of Tau$_{441}$ droplets in the absence and presence of Bri2 BRICHOS.*

Figure S12. *Monomeric Bri2 BRICHOS. Bri2 BRICHOS interacts with Tau$_{441}$ monomers in the droplet state.*

Figure S13. *γ-oscillation power in the presence or absence of 50 nM wildtype Tau$_{441}$ or Tau$_{P301L}$ monomers.*

Figure S14. *The Bri2 BRICHOS domain inhibits Tau$_{P301L}$ protein fibrillation and modulates the phase separation behavior.*

***References*…………………………………………………………………………………...**

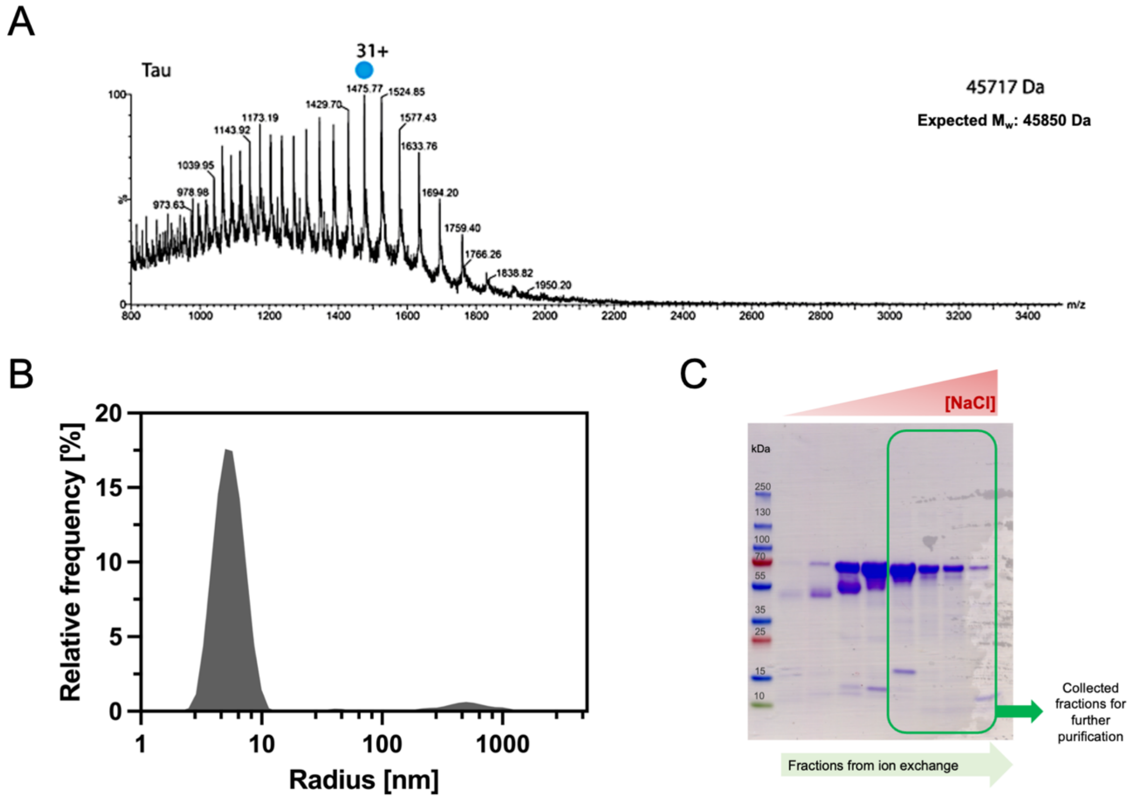

Figure S1. Recombinant Tau$_{441}$ protein quality control. (A) *Mass spectrum of Tau$_{441}$, the annotation [31+] corresponds to the highest charge state and is one example for the determination of the mass of Tau ((31x1475.77)-31=45718) and **(B)** Dynamic Light Scatterering (DLS) measurements of Tau$_{441}$, indicating a monomeric state of Tau$_{441}$. **(C)** SDS-PAGE of fractions collected from ion exchange during the purification process, indicating no higher molecular weight species of Tau$_{441}$. Only the purest fractions were used for the further purification steps.*

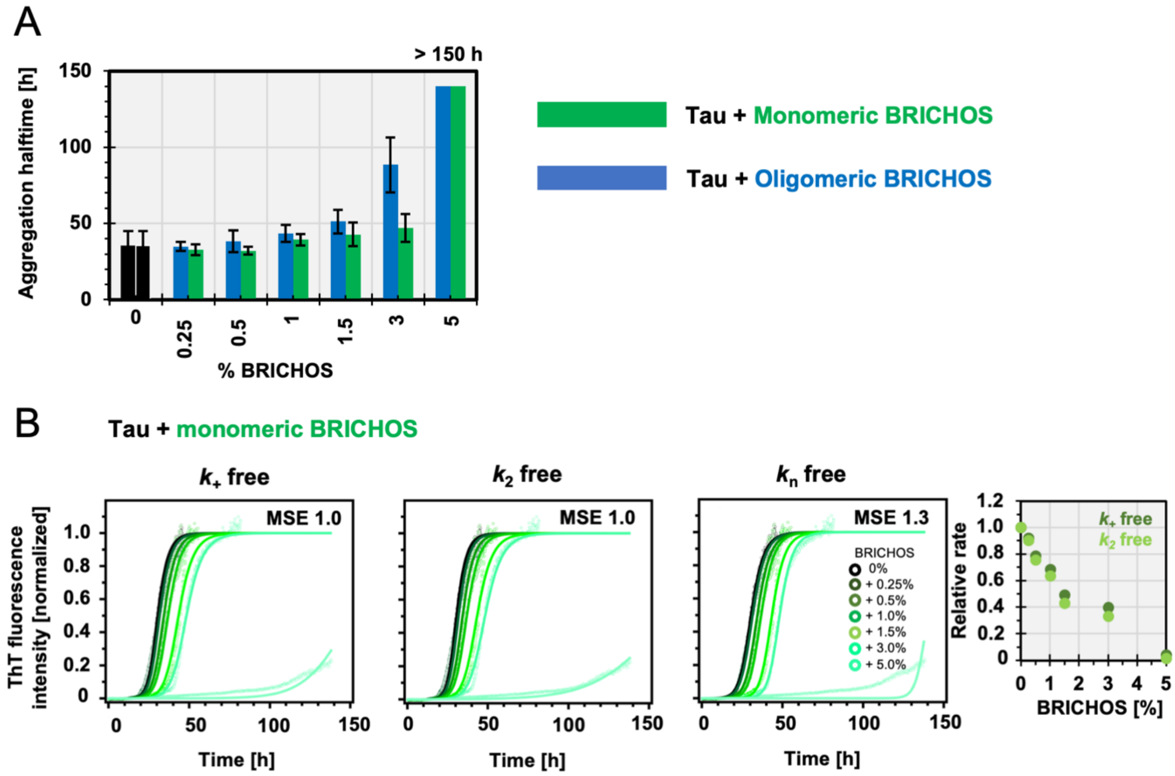

Figure S2. Cofactor-free Tau$_{441}$ aggregation is inhibited by both monomeric and oligomeric BRICHOS by inhibiting Tau$_{441}$ secondary nucleation processes. (A) *Aggregation halftimes determined by sigmoidal curvefitting of Thioflavin T (ThT) fluorescene intensity aggregation traces from samples with 20 µM full-length Tau$_{441}$ in 20 mM NaP buffer, pH 7.4, 0.2 mM EDTA, and 50 µM ThT during shaking conditions at +37 °C in the presence of monomeric or oligomeric Bri2 BRICHOS.* ***(B)*** *Global fit analysis of 20 µM full-length Tau$_{441}$ fibrillation in the presence of monomeric Bri2 BRICHOS. The data was fitted to a secondary nucleation dominated model using AmyloFit[1] where one nucleation rate was the free fitting parameter. The relative mean square error (MSE) values from the global fit analysis are stated. The relative nucleation rates from the global fit analysis are plotted against the relative Bri2 BRICHOS concentration in the right panel. Corresponding data utilizing oligomeric BRICHOS is presented in Figure 1 in the main manuscript.*

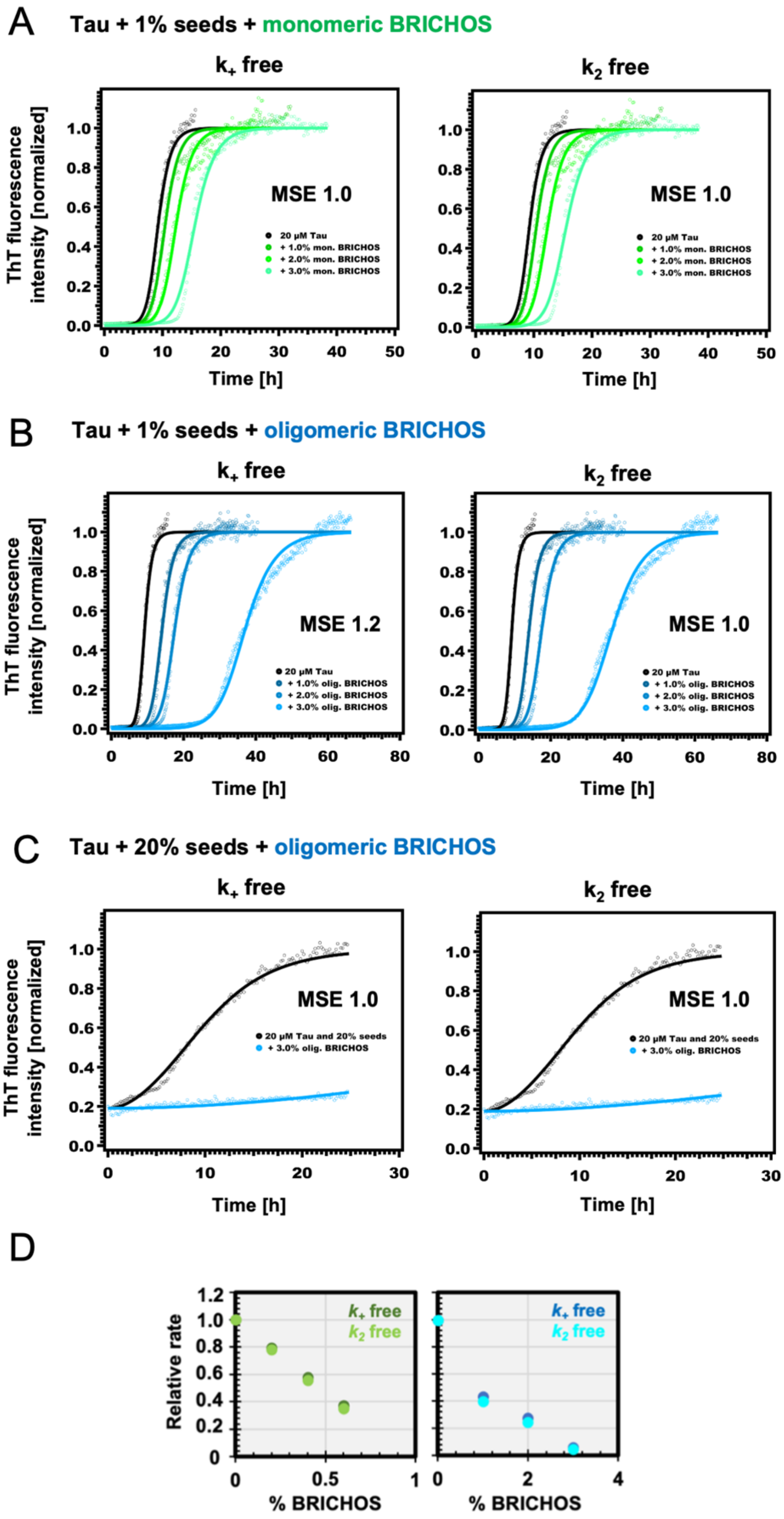

Figure S3. Bri2 BRICHOS inhibits Tau_{441} fibrillation both in the presence of 1% or 20% pre-formed seeds. *Global fit analysis of 20 µM full-length Tau_{441} fibrillation with 1%* ***(A, B)*** *or 20%* ***(C)*** *pre-formed Tau_{441} seeds in 20 mM NaP buffer, pH 7.4, and 50 µM ThT during shaking conditions at +37 °C in the presence of monomeric* ***(A)*** *or oligomeric Bri2 BRICHOS* ***(B, C)****. The global fit analysis was performed with a secondary nucleation dominated model using AmyloFit [1]. The relative mean square error (MSE) values are based on the global fit for comparison.* ***(D)*** *The relative nucleation rates for monomeric (green, left panel) and oligomeric (blue, right panel) Bri2 BRICHOS from the global fit analysis are plotted against the relative Bri2 BRICHOS concentration.*

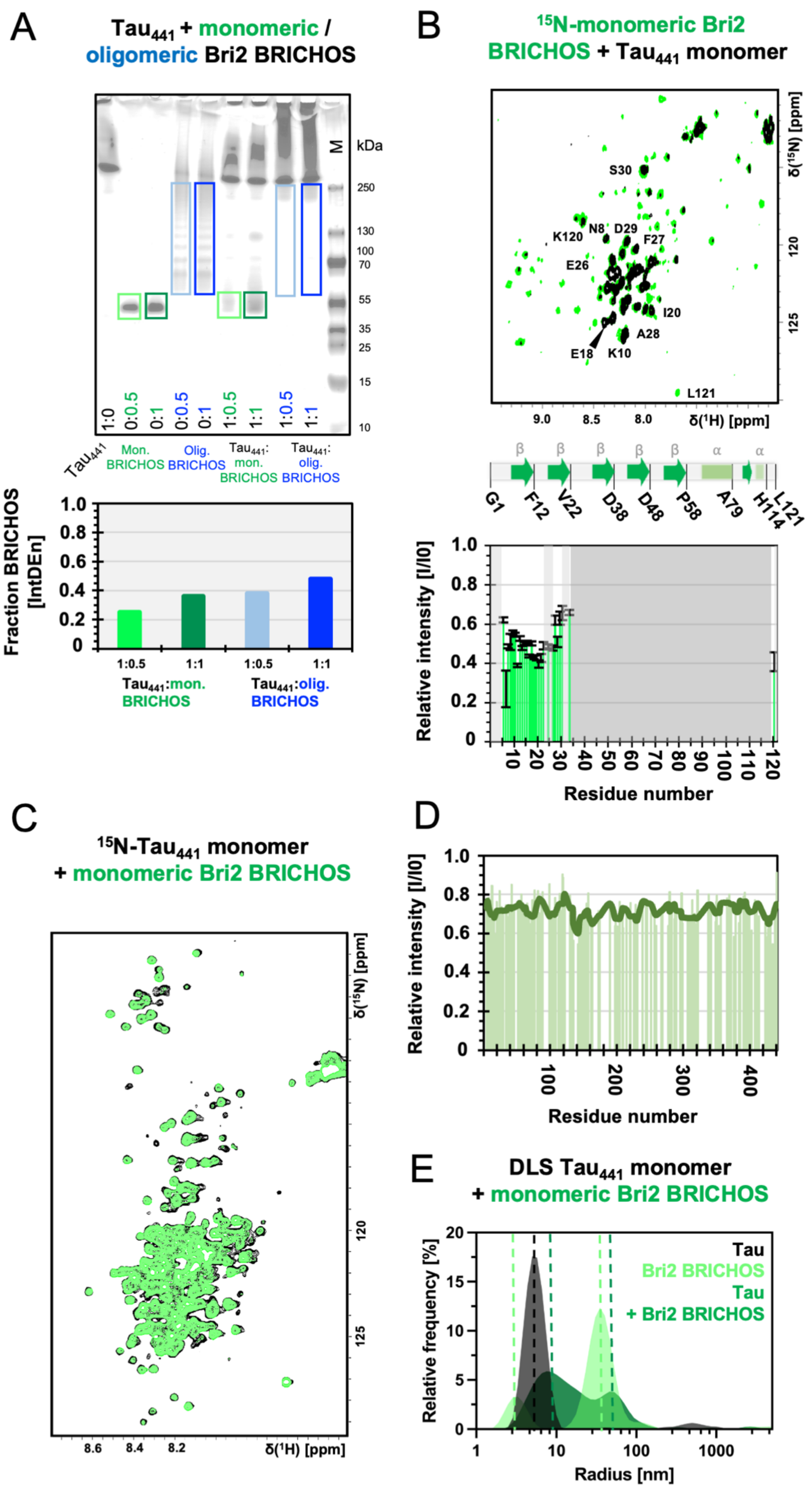

Figure S4. Monomeric Bri2 BRICHOS interacts with Tau₄₄₁ monomers. (A) *Native PAGE of Bri2 BRICHOS (12.5 µM or 25 µM) and 25 µM Tau$_{441}$ as mixtures. The intensity of the bands corresponding to Bri2 BRICHOS significantly decrease in the presence of Tau$_{441}$. Noticeably, the Tau samples exhibit a smear in the loading zone of the gel in addition to the more defined band, likely due to positive net charge of Tau. The conditions were not*

optimized for native PAGE of positively charged proteins, rather a standard gel and a setup for negatively charged proteins like BRICHOS were used to study the impact on BRICHOS. The marker is not optimized for native PAGE. The fractions of unbound Bri2 BRICHOS in the the presence of Tau441 were determined by comparing the intensities of the Bri2 BRICHOS bands with and without Tau441 using the Fiji software. ***(B)*** *^{1}H-^{15}N-HSQC experiments of 68 µM monomeric ^{15}N-labeled Bri2 BRICHOS (green) in the presence of 34 µM Tau441 protein (black). Due to unfavorable dynamics of Bri2 BRICHOS, only a small fraction of the residues, corresponding to the first ~ 30 N-terminal residues, is visible in the HSQC spectrum[2]. Residues marked with light grey shaded areas were excluded from the analysis due to low signal-to-noise ratio (<12), and the darker grey shaded areas represent residues not visible in the HSQC spectrum.* ***(C)*** *2D NMR ^{1}H-^{15}N-HSQC experiments with 100 µM ^{15}N-labeled monomeric Tau441 protein (black) in the presence of 100 µM monomeric (green) Bri2 BRICHOS. The NMR experiments were performed at 298 K in 20 mM NaP buffer, pH 7.4.* ***(D)*** *Relative intensities from the spectra in (C). Residues with a too low signal-to-noise ratio (<12) or overlap were excluded from the analysis. The solid line represents a smoothing function of 15 using the median.* ***(E)*** *DLS profiles of 25 µM Tau441 and 25 µM monomeric Bri2 BRICHOS, suggesting the formation of larger complexes for the Tau-Bri2 BRICHOS mixture. MTB: microtubule-binding domain. Corresponding data to (C) but utilizing oligomeric Bri2 BRICHOS is presented in Figure 2 in the main manuscript.*

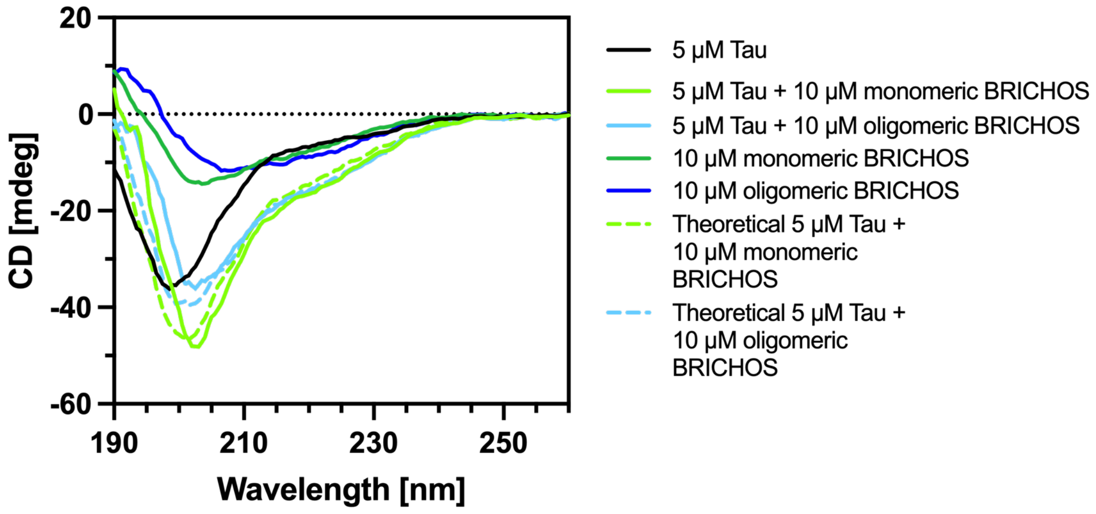

Figure S5. Secondary structures of Tau441 in the absence and presence of Bri2 BRICHOS using Circular Dichroism (CD) spectroscopy. *The secondary structures of Tau441 and Bri2 BRICHOS measured by CD spectroscopy in 20 mM NaP buffer, pH 7.4, and 0.2 mM EDTA at room temperature. The dashed spectra represent the theoretical spectrum when adding the individual spectra.*

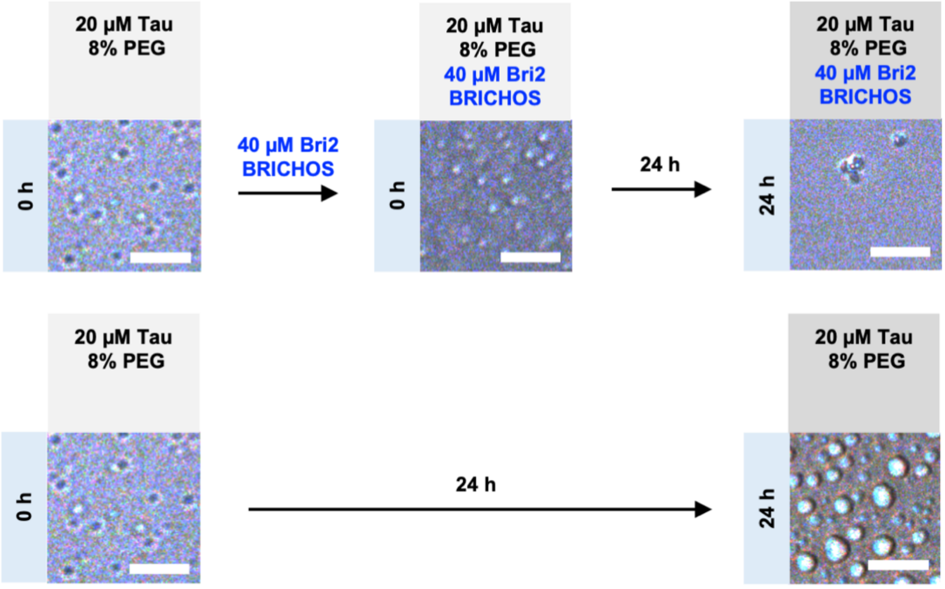

Figure S6. Bri2 BRICHOS dissolves pre-formed Tau$_{441}$ droplets after 24 hours compared to 0 h. *Tau droplets with 20 µM Tau$_{441}$ and 8% PEG8000 in 20 mM NaP buffer, pH 7.4, at room temperature were formed and Bri2 BRICHOS was subsequently added at 1:2 (Tau:Bri2 BRICHOS) molar ratio to the already formed and stable droplets. The samples were incubated for 24 h and brightfield microscopy images were recorded. The scale bar represents 10 µm.*

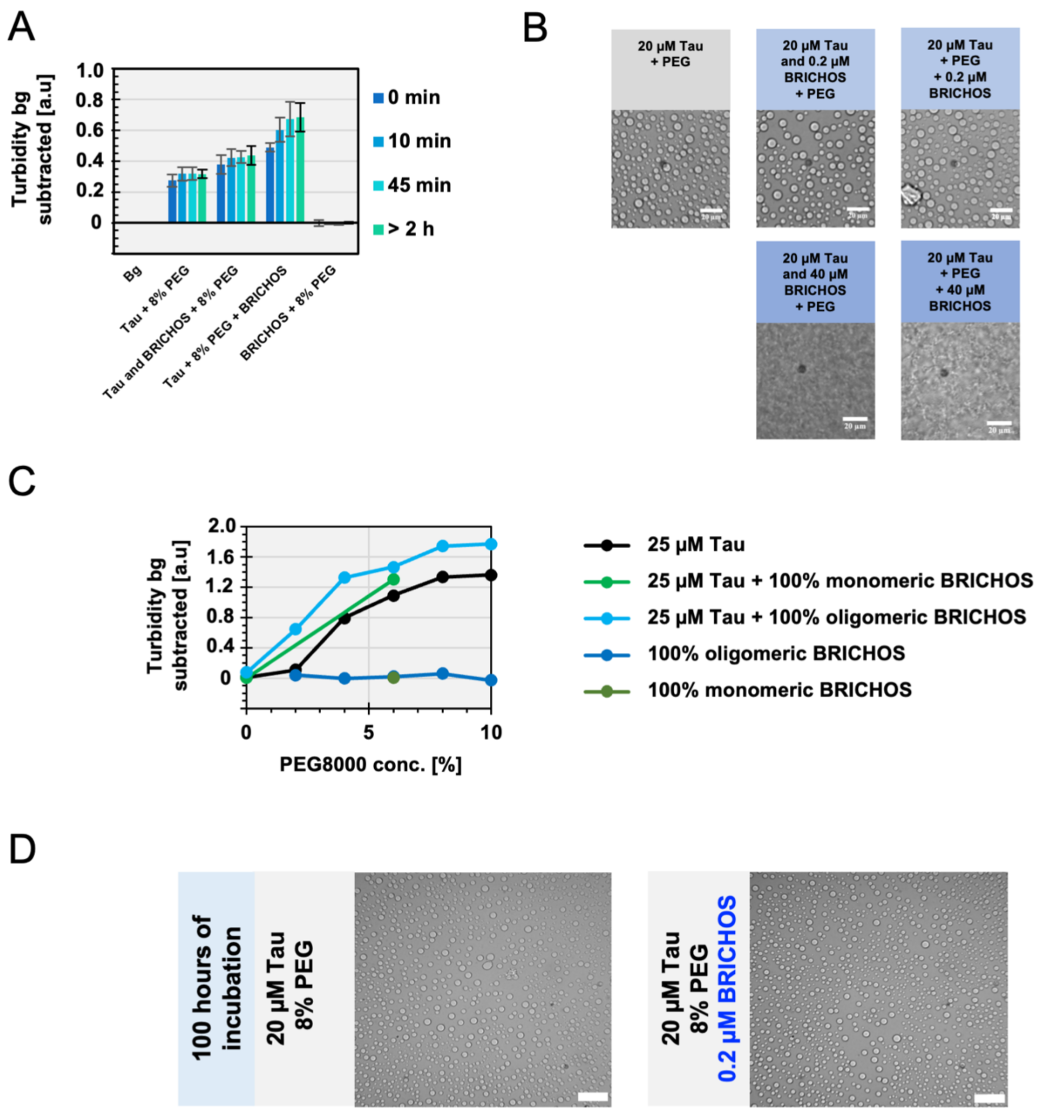

Figure S7. Tau$_{441}$ droplet formation using different Bri2 BRICHOS and PEG8000 concentrations and the time dependence. ***(A)*** *A mixture of 10 µM oligomeric Bri2 BRICHOS and 10 µM Tau$_{441}$ before addition of 8% (w/vol) PEG8000 display increased turbidity values compared to samples without Bri2 BRICHOS. Average and standard deviations values from three replicates are shown. When 10 µM oligomeric Bri2 BRICHOS was added to mixed samples of Tau$_{441}$ and PEG8000, the turbidity values were increased.* ***(B)*** *Under both conditions, similar droplets were observed at time zero and after 72 h. Experiments were performed under quiescent conditions, room temperature using 20 mM NaP, pH 7.4 buffer.* ***(C)*** *Turbidity values versus PEG8000 concentration for 25 µM Tau$_{441}$ in the absence and presence of 25 µM monomeric or oligomeric Bri2 BRICHOS.* ***(D)*** *DIC microscopy images of 20 µM Tau$_{441}$ and 8% PEG8000 in the absence or presence of 0.2 µM oligomeric Bri2 BRICHOS. The samples were incubated for 100 h. The scale bar represents 40 µm.*

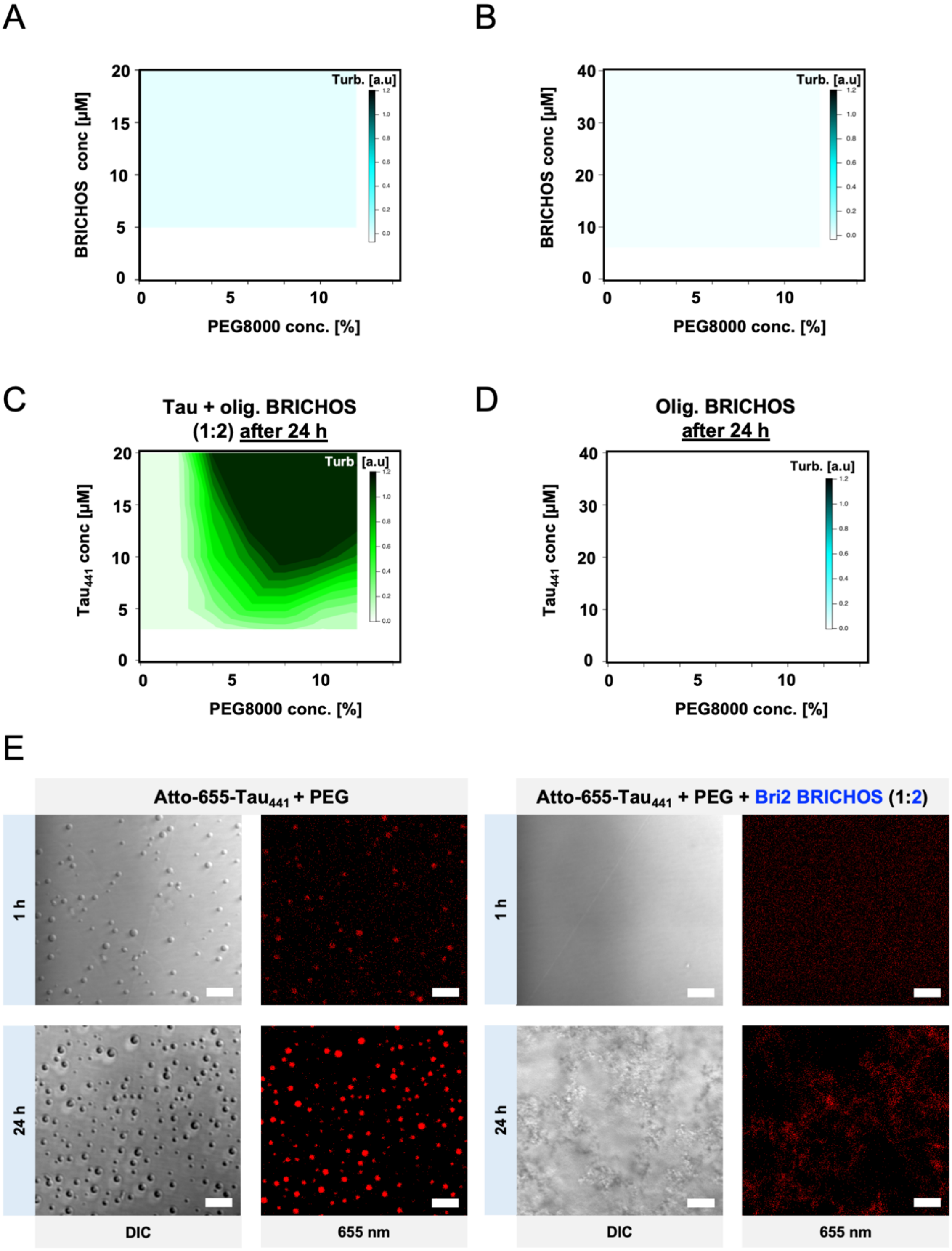

Figure S8. Microscopy images and phase diagrams of measured turbidity values for different Bri2 BRICHOS or Tau$_{441}$ protein and PEG8000 concentrations at different time points. ***(A, B)*** *Phase diagrams of measured turbidity values at 350 nm at varying oligomeric Bri2 BRICHOS protein and PEG8000 concentrations.* ***(C)*** *Phase diagram of measured turbidity values at varying protein and PEG8000 concentrations in the presence of super-stoichiometric concentrations of oligomeric Bri2 BRICHOS* ***after 24 h*** *(phase diagrams of fresh samples in the main manuscript). The same measurement as in* ***(C)*** *was performed with only oligomeric Bri2 BRICHOS and presented in* ***(D)****.* ***(E)*** *Brightfield and fluorescence microscopy images of 10 µM Atto-655-Tau$_{441}$ (1% labeled) in 8% PEG8000, 20 mM NaP, pH 7.4, at room temperature, in the absence or presence of 20 µM oligomeric Bri2 BRICHOS, at time points 1 and 24 hours. Droplets are observed for Tau$_{441}$, but are completely abolished in the presence of Bri2 BRICHOS. The microscopy images of Tau$_{441}$ and Bri2 BRICHOS after 24 hours show amorphous*

aggregates with increased fluorescent intensity from incorporated Atto-655-Tau441. The scale bar corresponsds to 10 µm.

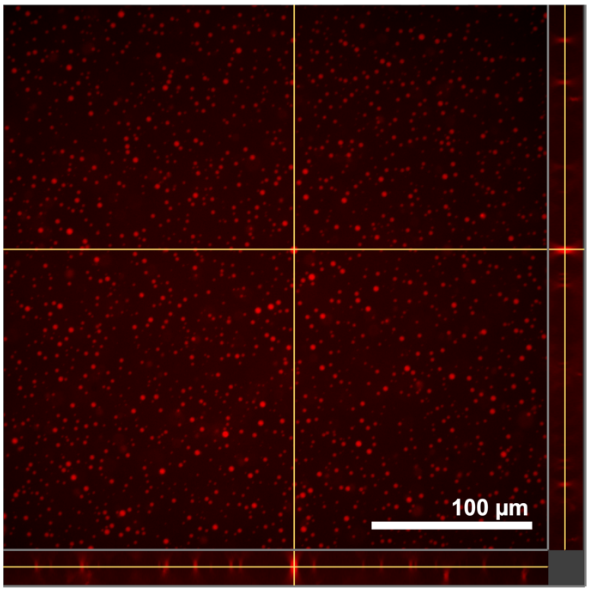

Figure S9. **Confocal microscopy Z-stack** ***image of Tau441 droplets with fluorescently labeled Bri2 BRICHOS.*** *The xy-stack at the level of the white/yellow lines show uniformly incorporated Bri2 BRICHOS. Fluorescence image of 20 µM Tau441 in the presence of 1% monomeric Bri2 BRICHOS fluorescently labeled with Atto-655 dye.*

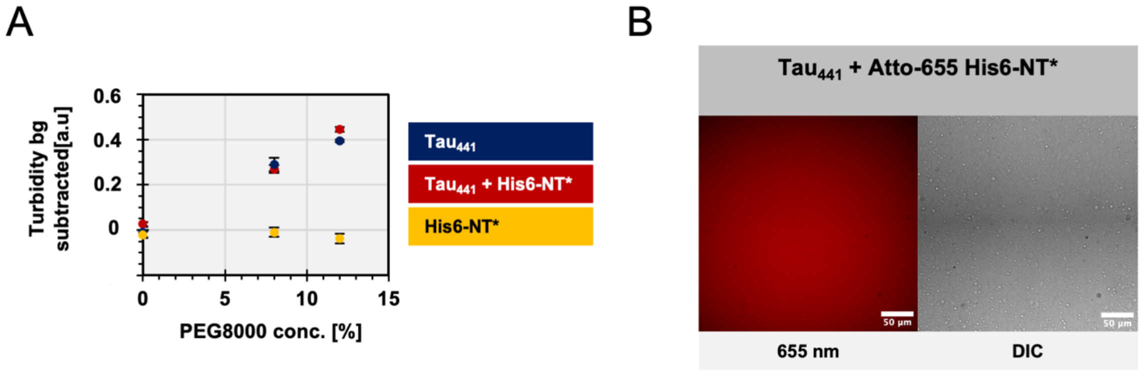

Figure S10. NT* (control protein) is not incorporated into Tau441 droplets. *To investigate any specificity for Bri2 BRICHOS towards Tau441 droplets we performed experiments with a control protein, NT* (reference[3]), of similar size as Bri2 BRICHOS. In* ***(A)*** *the turbidity values at 350 nm with different PEG8000 concentrations were measured, with 20 µM Tau441 (blue), 20 µM His6-NT* (yellow), and 20 µM Tau441 + 20 µM His6-NT* (red). His6-NT* did not induce any increase of the turbidity neither alone nor in the presence of Tau441 compared to Tau441 alone, indicating no influence on the droplet formation. Average and standard deviations values from N=3 replicates are shown.* ***(B)*** *Fluorescence microscopy was also measured with 0.2 µM Atto655-labeled NT* and 20 µM unlabeled Tau441 droplets with 8% PEG8000. No fluorescence increase was observed within the droplets, providing evidence of no incorporation into the droplets.*

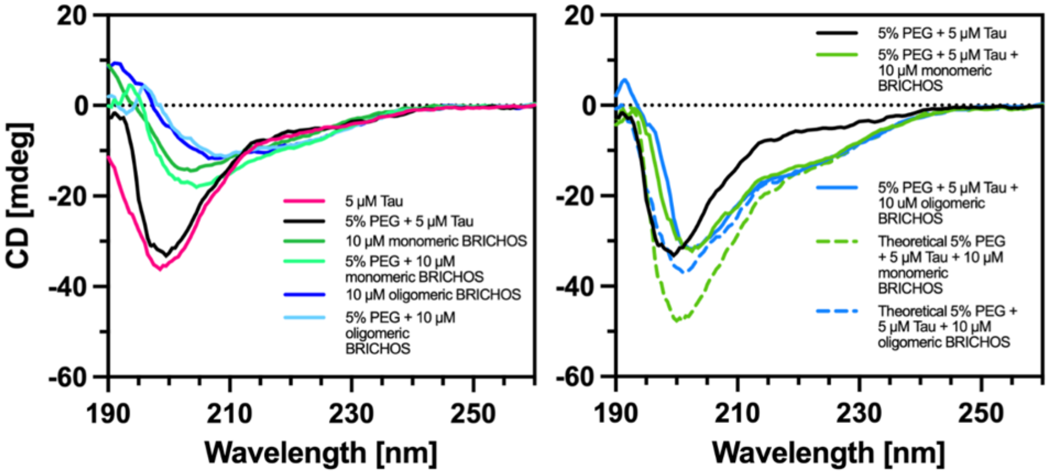

Figure S11. Secondary structures of Tau$_{441}$ droplets in the absence and presence of Bri2 BRICHOS. *5 µM Tau$_{441}$ protein with and without 5% PEG8000 in the presence and absence of either monomeric or oligomeric Bri2 BRICHOS in 20 mM NaP buffer, pH 7.4, at room temperature, using CD spectroscopy. The dashed spectra represent the theoretical spectrum when adding the individual spectra.*

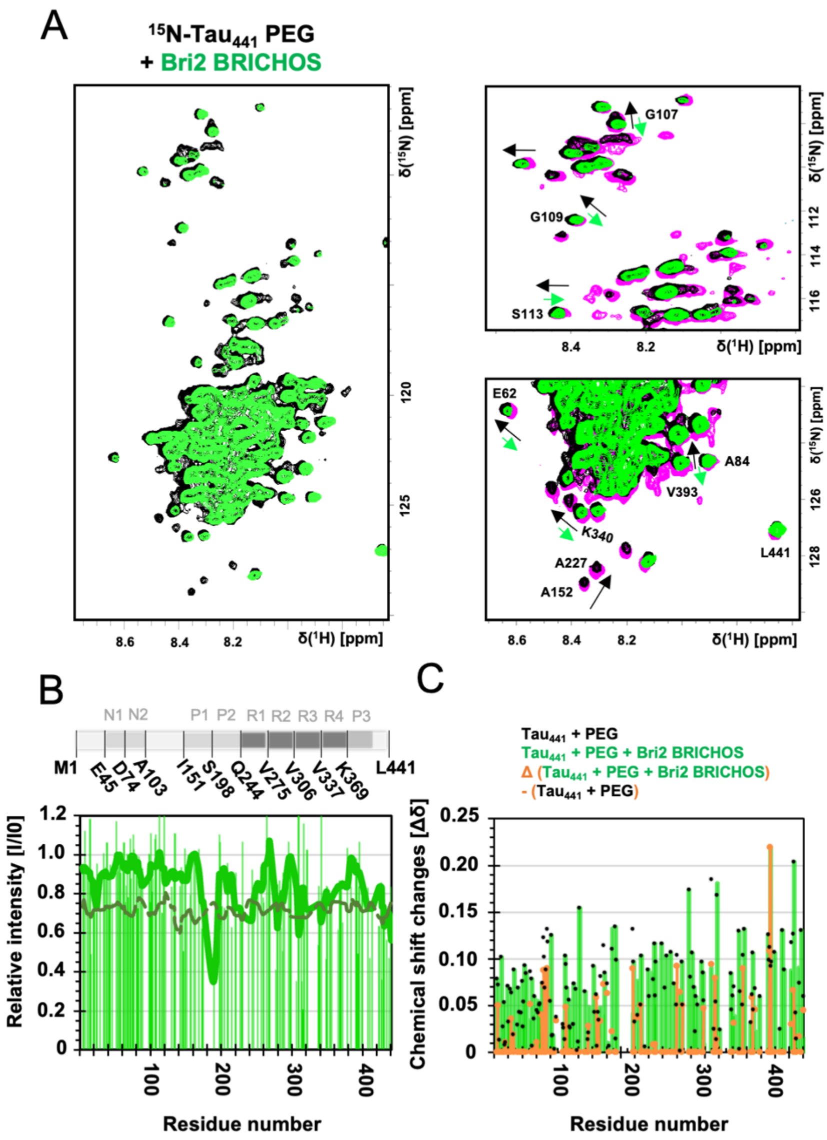

Figure S12. Monomeric Bri2 BRICHOS interacts with Tau$_{441}$ monomers in the droplet state. (A) *2D NMR ^{1}H-^{15}N-HSQC experiments with final concentrations 62 µM ^{15}N-labeled monomeric Tau$_{441}$ protein (pink) and addition of 6% PEG8000 (black) and 62 µM monomeric Bri2 BRICHOS (green). The experiments were performed at 298 K in 20 mM NaP buffer, pH 7.4, 0.2 mM EDTA (90/10 H_2O/D_2O). The information from the spectra were analyzed and the signal intensity change and the chemical shift changes are presented in **(B)** and **(C)**, respectively. The solid line in **(B)** represents a smoothing function of 15 using the median, and the dashed line corresponds to data without PEG from Figure 2 for ^{15}N-Tau$_{441}$ and monomeric Bri2 BRICHOS. In **(C)**, the chemical shift changes for Tau$_{441}$ and PEG8000 (black dots), and Tau$_{441}$, PEG8000 and monomeric Bri2 BRICHOS (green bars), and the difference (orange), showing that the presence of Bri2 BRICHOS (green bars) counteracts the induced chemical shift changes by PEG8000 (black dots) for most residues. MTB: microtubule-binding domain. Corresponding data utilizing oligomeric BRICHOS is presented in Figure 4 in the main manuscript.*

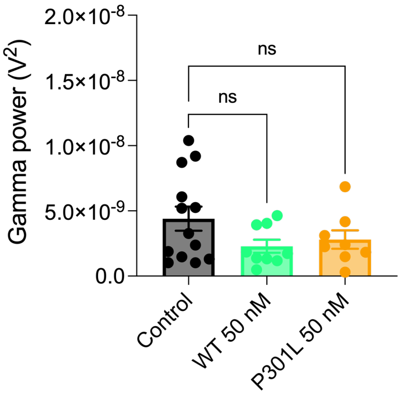

Figure S13. γ-oscillation power in the presence or absence of 50 nM wildtype Tau$_{441}$ or Tau$_{P301L}$ monomers. *γ-oscillation power in the presence or absence of 50 nM wildtype Tau$_{441}$ (green, N=9) or Tau$_{P301L}$ monomers (beige, N=8) revealing no significant impact. The data are expressed as the mean ± SEM, and the statistical significance was estimated by one-way ANOVA, followed by Tukey's multiple comparisons test. *p < 0.05, **p < 0.01, ns, not significant.*

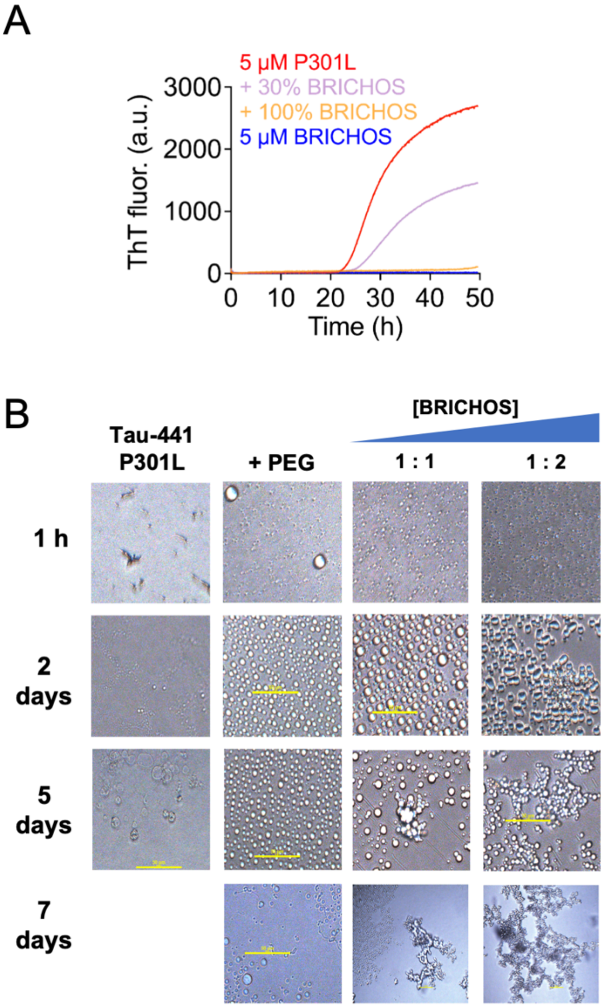

Figure S14. Bri2 BRICHOS inhibits Tau$_{P301L}$ fibrillation and modulates Tau$_{P301L}$ phase separation behavior. ***(A)*** *Thioflavin T (ThT) fluorescence kinetics of 5 µM full-length Tau$_{P301L}$ in the presence of 0, 30, and 100% monomeric Bri2 BRICHOS, supplemented with ThT and heparin in NaP buffer.* ***(B)*** *Brightfield microscopy images of 10 µM full-length Tau$_{P301L}$, 8% PEG8000 in 20 mM NaP buffer, pH 7.4, at room temperature, in the presence of different concentrations of oligomeric Bri2 BRICHOS, at four different time points. The droplet formation propensities are increased in the presence of 1:1 ratio but are compromised above equimolar Bri2 BRICHOS concentrations.*

References

1. Meisl, G. *et al.* Molecular mechanisms of protein aggregation from global fitting of kinetic models. *Nat Protoc* **11**, (2016).
2. Adam, L. *et al.* Specific inhibition of α-synuclein oligomer generation and toxicity by the chaperone domain Bri2 BRICHOS. *Protein Science* **33**, e5091 (2024).
3. Kronqvist, N. *et al.* Efficient protein production inspired by how spiders make silk. *Nat Commun* **8**, (2017).
